# Supplementary material for: The centrosomal protein 131 participates in the regulation of mitochondrial apoptosis
Source: Commun Biol. 2023 Dec 15;6:1271. doi: 10.1038/s42003-023-05676-3 (PMC10724242; doi:10.1038/s42003-023-05676-3)
Supplement: Supplementary file 2 — Supplemental material [file 42003_2023_5676_MOESM2_ESM.pdf]

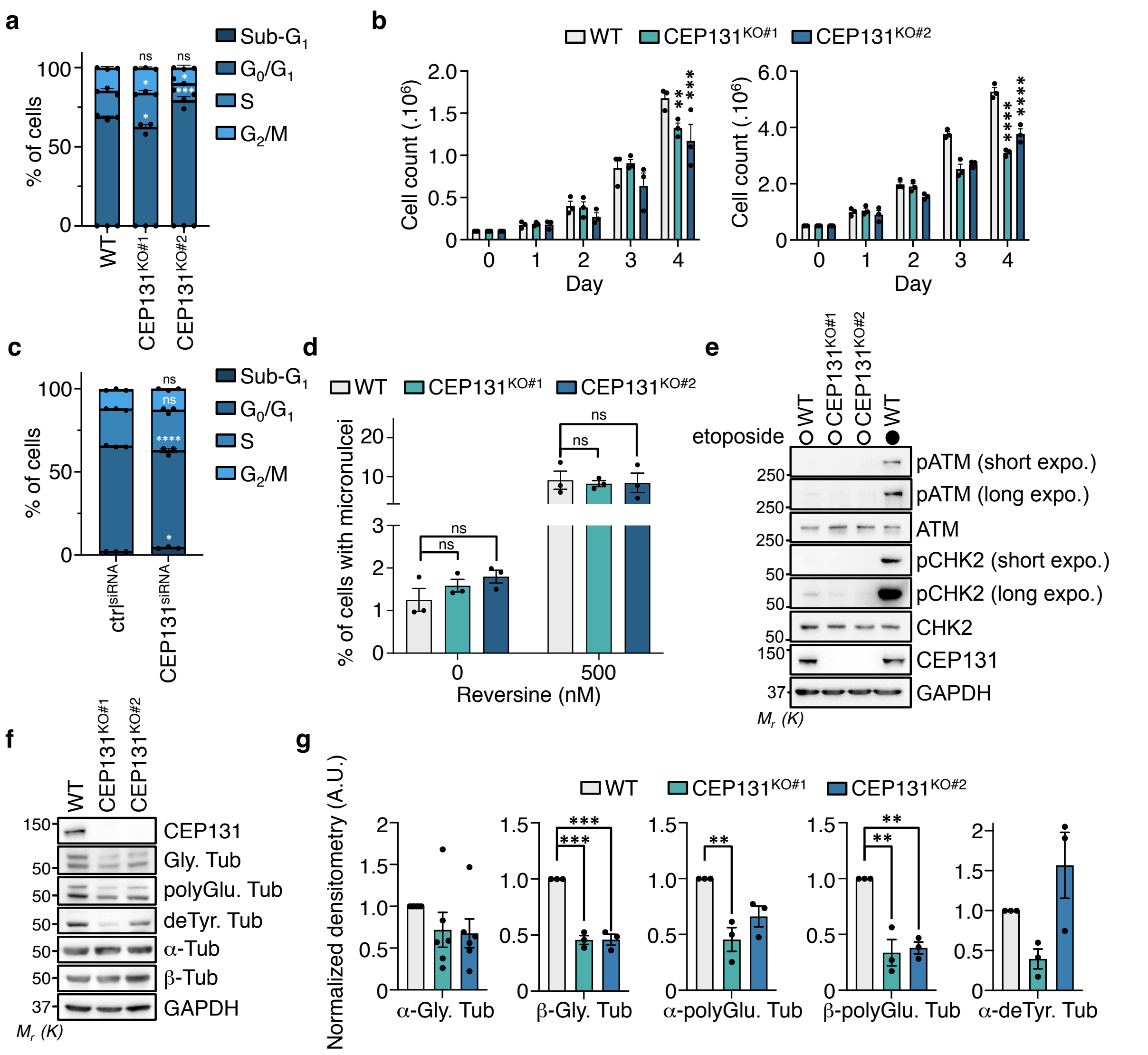

Supplementary Fig. 1 – Characterization of CEP131 knockout Jurkat Cells, related to Fig. 1

(a) Cell cycle distribution was assessed in wild-type (WT), CEP131KO#1, and KO#2 Jurkat (mean ± SEM, n=3 biological replicates, two-way ANOVA, *p<0.05, ***p<0.001). **(b)** WT, CEP131KO#1, and KO#2 Jurkat cells were plated at $0.1.10^6$ $cells.mL^{-1}$ (left) or $0.5.10^6$ $cells.mL^{-1}$ (right) at day 0 and were counted each day as indicated (mean ± SEM, n=3 biological replicates, two-way ANOVA, **p<0.01, ***p<0.001, ****p<0.0001). **(c)** L929 cells were transfected with siRNA targeting CEP131 ($CEP131^{siRNA\#1}$) or with non-specific control siRNA ($ctrl^{siRNA}$) for 72h, and cell cycle was assessed (mean ± SEM, n=3 biological replicates, two-way ANOVA, *p<0.05, ****p<0.0001). **(d)** WT, CEP131KO#1, and KO#2 Jurkat cells were treated or not for 48h with 500 nM reversine. Cells were stained with DAPI, and micronuclei were counted (n=200 cells/condition, mean ± SEM, n=3 biological replicates, two-way ANOVA, ns: non-significant). **(e)** Cell lysates from WT, CEP131 KO#1, and KO#2 cells stimulated or not with 5 µM etoposide for 1h were prepared and analyzed by immunoblotting with specific antibodies. **(f and g)** Cell lysates from WT, CEP131 KO#1, and KO#2 cells were prepared and analyzed by immunoblotting with specific antibodies (e). The densitometric analyses were performed and normalized with a loading control (f). WT values were set to 1 (mean ± SEM, n=3 or 6 biological replicates, one-way ANOVA; **p<0.01, ***p<0.001).

Data information: **(e and f)** GAPDH, α-Tubulin, or β-Tubulin served as loading controls. Molecular weight markers are shown. Data are representative of three independent experiments.

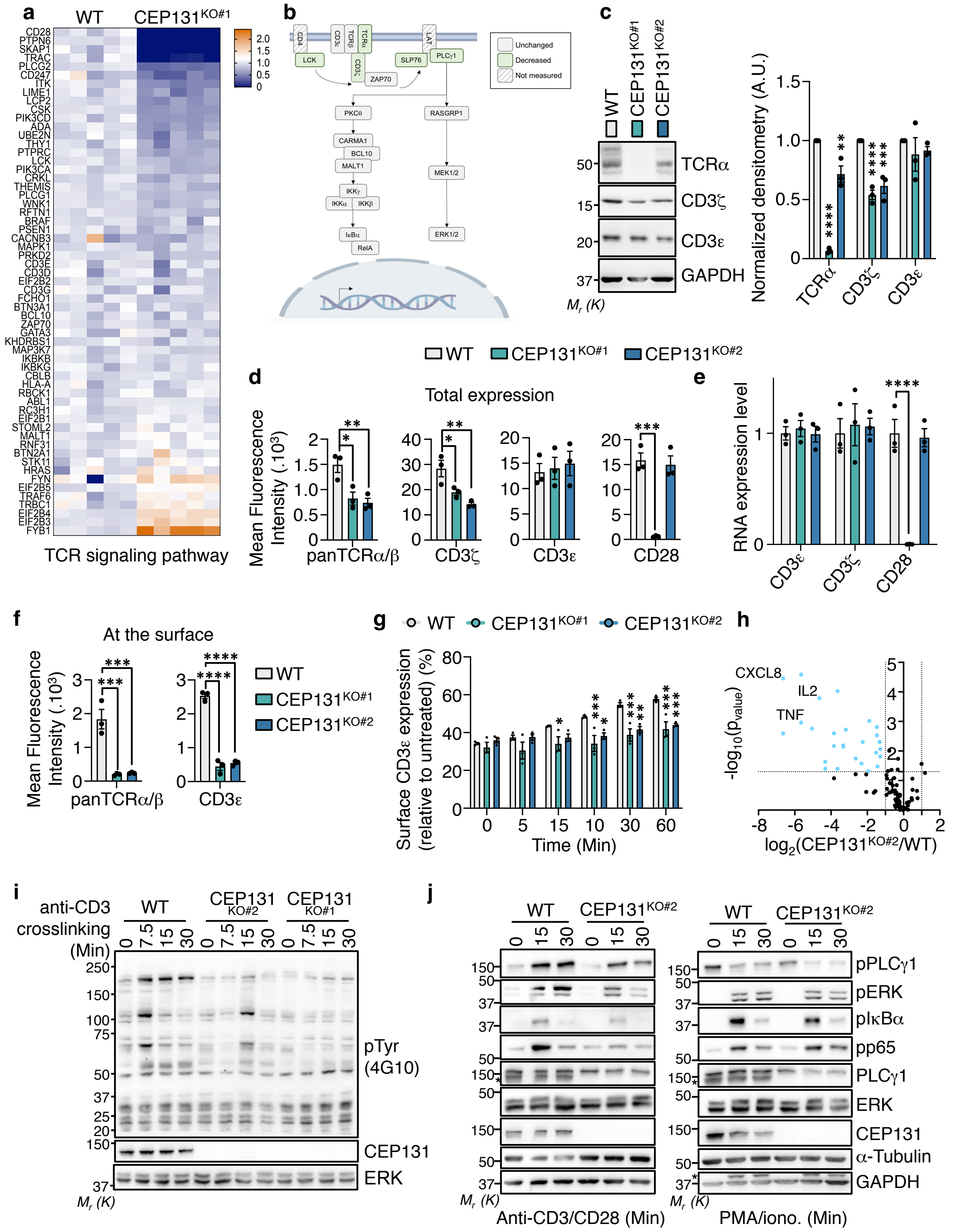

Supplementary Fig. 2 – CEP131 regulates the T-cell Receptor Pathway

(a) Heatmap of the normalized abundance of TCR signaling components (GO:0050852) in CEP131 KO#1 Jurkat compared to wild-type (WT) cells based on proteomic analysis (n=5 replicates). **(b)** Schematic of TCR signaling pathway. In green are the proteins downregulated in CEP131 KO#1 cells. **(c)** Cell lysates from WT, CEP131 KO#1, and KO#2 Jurkat cells were prepared and analyzed by immunoblotting with antibodies specific to the indicated proteins. A densitometric analysis was performed and normalized with GAPDH. WT values were set to 1 (mean ± SEM, n=3 biological replicates, two-way ANOVA, **p<0.01, ***p<0.001,

****p<0.0001). **(d)** Flow cytometry analysis of the total expression of panTCRα/β, CD3ζ, CD3ε, and CD28 in WT, CEP131 KO#1 and KO#2 cells (mean ± SEM, n=3 biological replicates, one-way ANOVA, *p<0.05, **p<0.01, ***p<0.001). Note that CD28 expression is lost in CEP131 KO#1. **(e)** The RNA expression levels of CD3ε, CD3ζ, and CD28 were assessed by qPCR in WT, CEP131 KO#1, and KO#2 cells (mean ± SEM, n=3 biological replicates, fold change using ACTB and HPRT1 as housekeeping genes for normalization, two-way ANOVA, ****p<0.0001). **(f)** Flow cytometry analysis of the surface expression of panTCRα/β and CD3ε in WT, CEP131KO#1, and KO#2 cells (mean ± SEM, n=3 biological replicates, two-way ANOVA, ***p<0.001, ****p<0.0001). **(g)** Cells were treated with 1 µM Phorbol 12,13-dibutyrate (PDBu) for 1h and then washed and incubated at 37°C for the indicated times. Surface expression of CD3ε in WT, CEP131KO#1, and KO#2 cells relative to untreated was assessed by flow cytometry (mean ± SD, n=3 biological replicates, two-way ANOVA, ***p<0.001, ****p<0.0001). **(h)** Volcano plot of RT^2 profiler PCR array of human NF-κB signaling targets (Supplementary Table 2) for cells stimulated for 2h with 1 $\mu g.mL^{-1}$ of anti-CD3 and anti-CD28. Genes in blue are significantly downregulated (FC>2) in $CEP131^{KO\#2}$ cells compared with WT cells (mean of n=3 biological replicates). **(i)** WT and CEP131 KO#2 cells were stimulated with 10 $\mu g.mL^{-1}$ of mouse anti-CD3 for 20 min and then placed at 37°C with 5 $\mu g.mL^{-1}$ of goat anti-mouse for the indicated times. Cell lysates were prepared and analyzed by immunoblotting with antibodies specific to the indicated proteins. **(j)** Cells, as in (i) were stimulated with 1 $\mu g.mL^{-1}$ of anti-CD3 and anti-CD28 (left) or 20 $ng.mL^{-1}$ of Phorbol 12-myristate 13-acetate (PMA) and 300 $ng.mL^{-1}$ of ionomycin (right) for the indicated times. Cell lysates were prepared and analyzed by immunoblotting with antibodies specific to the indicated proteins.
Data information: **(c, i, j)** GAPDH or ERK served as loading controls. Molecular weight markers (M_r) are shown. Data are representative of three independent experiments.

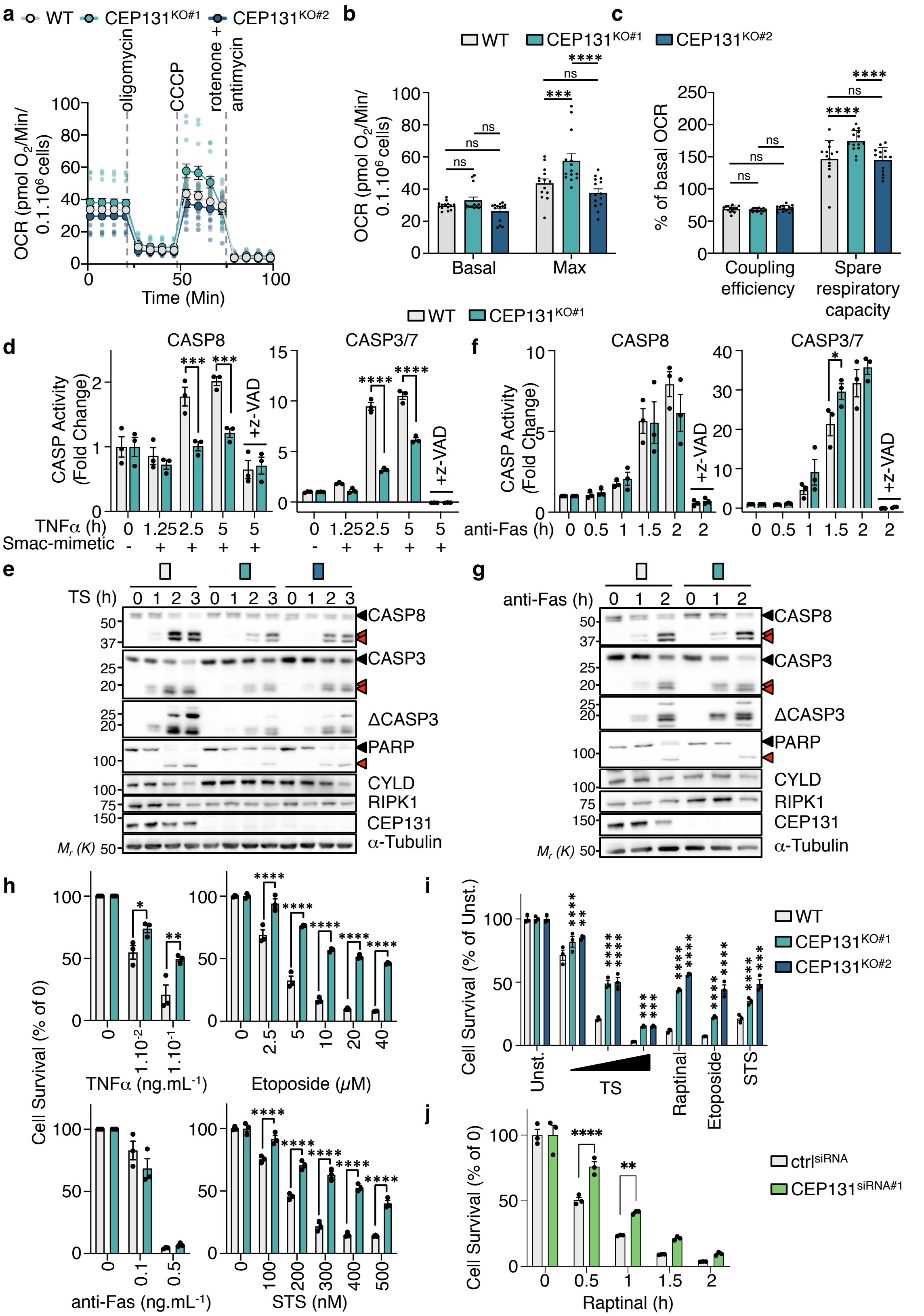

Supplementary Fig. 3. CEP131 participates in Mitochondria-dependent Cell Death induced by multiple Stimuli

(a-c) Metabolic profile of wild-type (WT), CEP131 KO#1, and KO#2 Jurkat cells using the Seahorse technology. Mitochondrial respiration overtime **(a)**, basal and maximal respiration **(b),** and coupling efficiency and spare capacity **(c)** are presented. (mean ± SEM, n = 14 replicates from 3 independent experiments, two-way ANOVA, ***p<0.001, ****p<0.0001).

(d) CASP8 and CASP3/CASP7 catalytic activity was measured by luminescent substrate cleavage (Caspase-Glo assay) in cells treated with 10 ng.mL^{-1} TNFα for the indicated times in the presence of 5 μM Birinapant (Smac-mimetic). The pan-caspase inhibitor z-VAD (20 μM) served as a control. (mean ± SEM, n=3 replicates, one experiment representative of three independent ones is shown, two-way ANOVA, ***p<0.001, ****p<0.0001). **(e)** Cell lysates from WT, CEP131KO#1, and KO#2 cells stimulated as in (d) for the indicated times were analyzed by immunoblotting with antibodies specific to the indicated proteins. **(f)** CASP8 and CASP3/CASP7 catalytic activity as in (d) in cells treated with 1 ng.mL^{-1} anti-Fas plus 1 ng.mL^{-1} protein A for the indicated times (mean ± SEM, n=3 biological replicates, two-way ANOVA, *p<0.05). **(g)** Cell lysates from WT and CEP131KO#1 stimulated as in (f) for the indicated times were analyzed by immunoblotting with antibodies specific to the indicated proteins. **(h)** Cell viability assessed by CellTiter Glo in cells treated with increasing concentrations of TNFα plus Birinapant, etoposide, staurosporine (STS), or anti-Fas (as indicated) for 16h in WT and CEP131 KO#1 (mean ± SEM, n= 3 replicates, two-way ANOVA, *p<0.05, **p<0.01, ****p<0.0001). **(i)** Cell viability was assessed as in (h) after treatment with increasing doses of TNFα and Birinapant (TS), 1 μM Raptinal, 10 μM etoposide, or 400 nM staurosporine (STS) for 16h (mean ± SEM, n= 3 replicates, one experiment representative of three independent ones is shown, two-way ANOVA, **p<0.01, ***p<0.001, ****p<0.0001). **(j)** L929 cells were transfected with siRNA targeting CEP131 (CEP131$^{siRNA\#1}$) or with non-specific control siRNA (ctrl^{siRNA}) for 72h. Cells were treated with 10 μM Raptinal for the indicated times. Cell viability was assessed by CellTiter Glo (mean ± SEM, n=3 replicates, one experiment representative of three independent ones is shown, two-way ANOVA, **p<0.01, ***p<0.0001).

Data information: **(e and g)** Full-length and cleaved protein forms are indicated with black and red arrowheads, respectively. α-Tubulin served as a loading control. Molecular weight markers (M_r) are shown. Data are representative of three independent experiments.

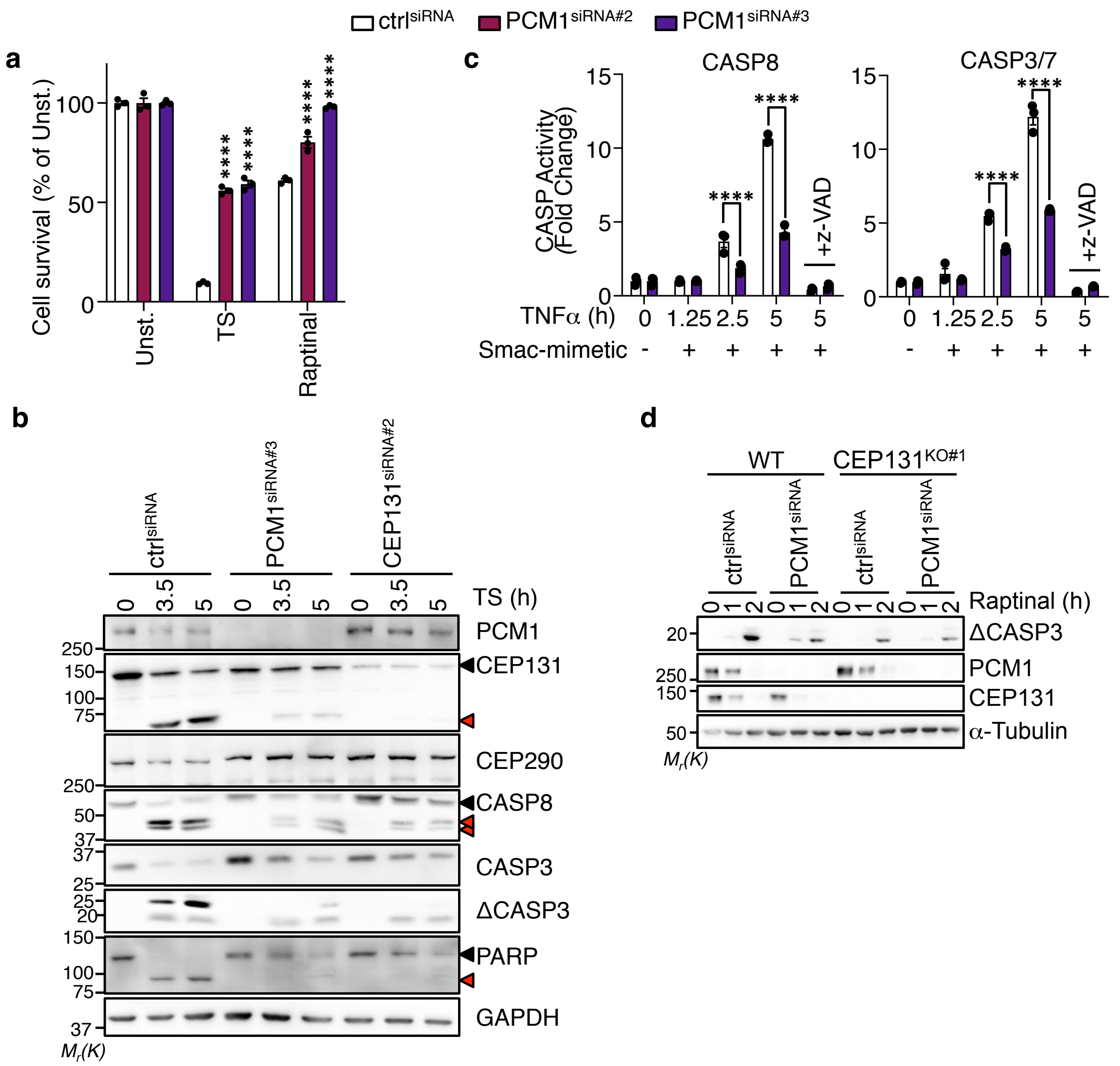

Supplementary Fig. 4. Silencing of PCM1 recapitulates CEP131 knockout Phenotype
(a) HT-29 cells were transfected with siRNA targeting PCM1 (PCM1$^{siRNA\#2}$ or PCM1$^{siRNA\#3}$) or with non-specific control siRNA (ctrl^{siRNA}) for 72h. Cells were pre-treated with 5 μM Birinapant (S) and stimulated with 1 ng.mL^{-1} TNFα (T) or with 5 μM Raptinal for 16h. Cell viability was assessed by CellTiter Glo (mean ± SEM, n=3 replicates, one experiment representative of three independent ones is shown, two-way ANOVA, ****p<0.0001). **(b)** HT-29 cells were transfected with siRNA targeting PCM1 (PCM1$^{siRNA\#3}$), CEP131 (CEP131$^{siRNA\#2}$), or non-specific control siRNA (ctrl^{siRNA}) for 72 h. Cells were pre-treated with 5 μM Birinapant (S) and stimulated with 10 ng.mL^{-1} TNFα (T) for the indicated times. Cell lysates were prepared and analyzed by immunoblotting with antibodies specific to the indicated proteins. **(c)** CASP8 and CASP3/CASP7 catalytic activity was measured by luminescent substrate cleavage (Caspase-Glo assay) in cells treated with 10 ng.mL^{-1} TNFα (T) (pre-treated with 5 μM Birinapant (S) and 20 μM z-VAD) for the indicated times (mean ± SEM, n=3 replicates, one experiment representative of three independent ones is shown, ANOVA, ****p<0.0001). **(d)** WT and CEP131KO#1 Jurkat cells were transfected with siRNA targeting PCM1 (PCM1$^{siRNA\#3}$) and were stimulated with 10 μM Raptinal for the indicated times. Cell lysates were prepared and analyzed by immunoblotting with antibodies specific to the indicated proteins.
Data information: **(b and d)** Full-length and cleaved protein forms are indicated with black

and red arrowheads, respectively. GAPDH or α-Tubulin served as loading controls. Molecular weight markers (M_r) are shown. Data are representative of three independent experiments.

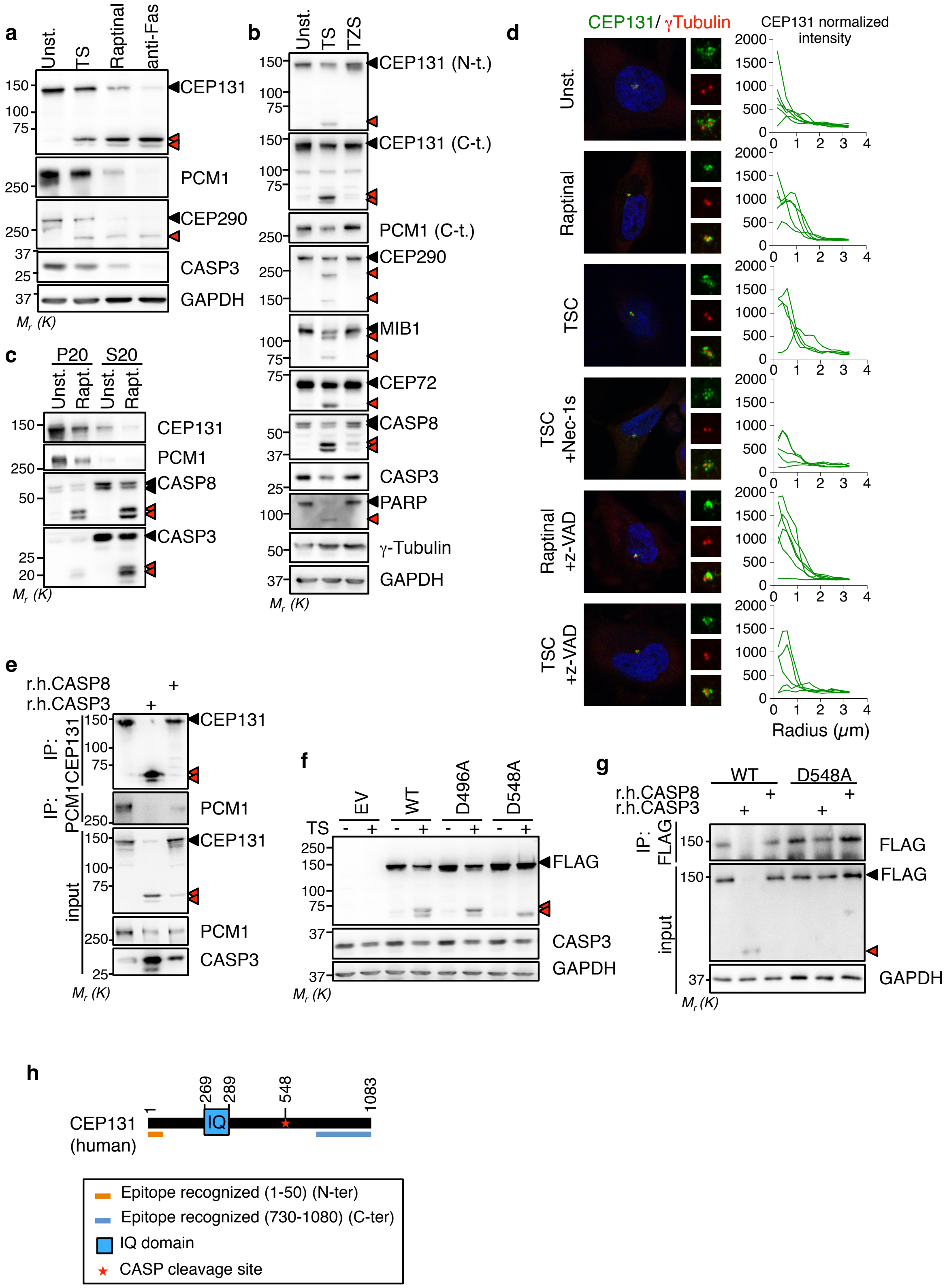

Supplementary Fig. 5. Centriolar Satellite Components are remodeled during Cell Death

(a) Jurkat cells were stimulated with 10 ng.mL^{-1} TNFα (T) in presence of 5 µM Birinapant (Smac-mimetic, S), 5 µM Raptinal, or 100 ng.mL^{-1} anti-Fas plus Protein A for 4h. Cell lysates were prepared and analyzed by immunoblotting with antibodies against the indicated proteins.

(b) Immunoblotting analysis of cell lysates from Jurkat cells stimulated as in (a). When indicated, 20 µM of the pan-caspase inhibitor (zVAD, Z) was used. **(c)** Jurkat cells were treated or not with 10 µM Raptinal for 2h. Fractions enriched with centriolar satellites (P20) and with cytosol (S20) were analyzed by immunoblotting with antibodies against the indicated proteins. **(d)** Confocal microscopy analysis of CEP131 (green) and γ-tubulin (red) in HeLa cells treated with 10 $ng.mL^{-1}$ TNFα (T) in presence of 5 µM Birinapant (Smac-mimetic, S) and 1 µM cycloheximide (C), or with 10 µM Raptinal for 7h. The RIPK1 inhibitor Nec-1s (Necrostatine-1s, 20 µM) or the pan-caspase inhibitor z-VAD (20 µM) were also used. The radial profile analysis was used to measure the dispersion of CEP131 around the centrosome (n= at least 4 cells/cond.). Nuclei were counterstained with 4'-6-diamidino-2- phenylindole (DAPI). Scale bars, 10 µm. Representative images are shown. **(e)** CEP131 and PCM1, immunopurified from Jurkat lysates, were incubated with recombinant human CASP3 or CASP8 for 1h at 37°C. Samples were then analyzed by immunoblotting as indicated. **(f)** Jurkat cells were transfected with plasmids encoding for CEP131-FLAG WT, CEP131-FLAG mutants (D496A or D548A), or with an empty vector (EV). Cells were then treated with 10 $ng.mL^{-1}$ TNFα (T) in presence of 5 µM Birinapant (Smac-mimetic, S) for 3h. Cell lysates were prepared and analyzed by immunoblotting with antibodies against the indicated proteins. **(g)** CEP131-FLAG WT or CEP131-FLAG mutant (D548A) were immunopurified from cells as in (f) prior to incubation with recombinant human CASP3 or CASP8 for 1h at 37°C. Samples were then analyzed by immunoblotting as indicated. **(h)** Schematic of cleavage site identified in human CEP131.
Data information: **(a, b, c, e, f, g)** Full-length and cleaved protein forms are indicated with black and red arrowheads, respectively. Molecular weight markers (M_r) are shown. Data are representative of three independent experiments**. (a, b, f, g)** GAPDH served as a loading control.

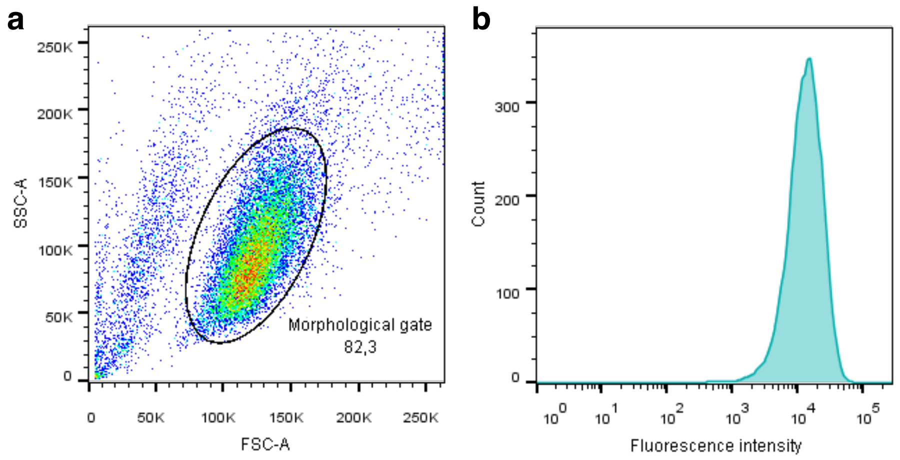

Supplementary Fig. 6. Details of the flow cytometry gating strategy.
a and b, Cells were gated with FSC and SSC parameters, and histogram analyses were performed as indicated in the figure legends.

Supplementary Fig. 7. Uncropped blots.

Figure 1c

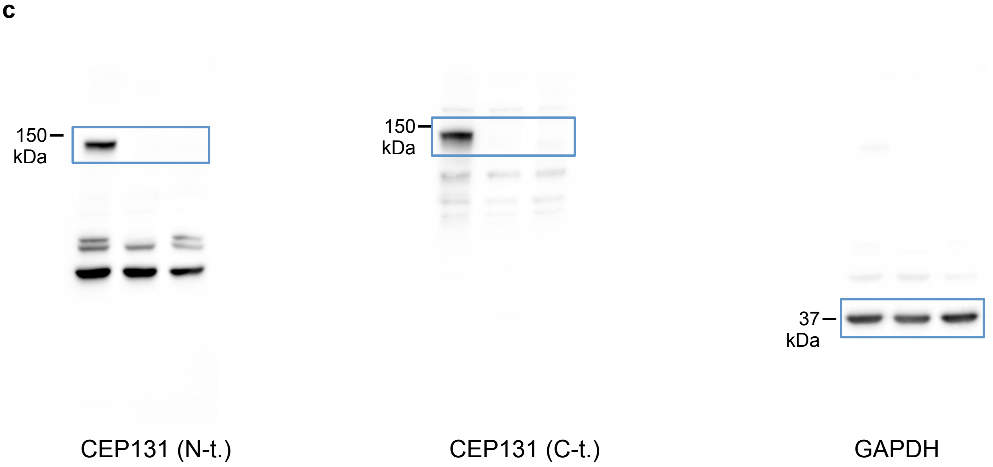

Figure 1d

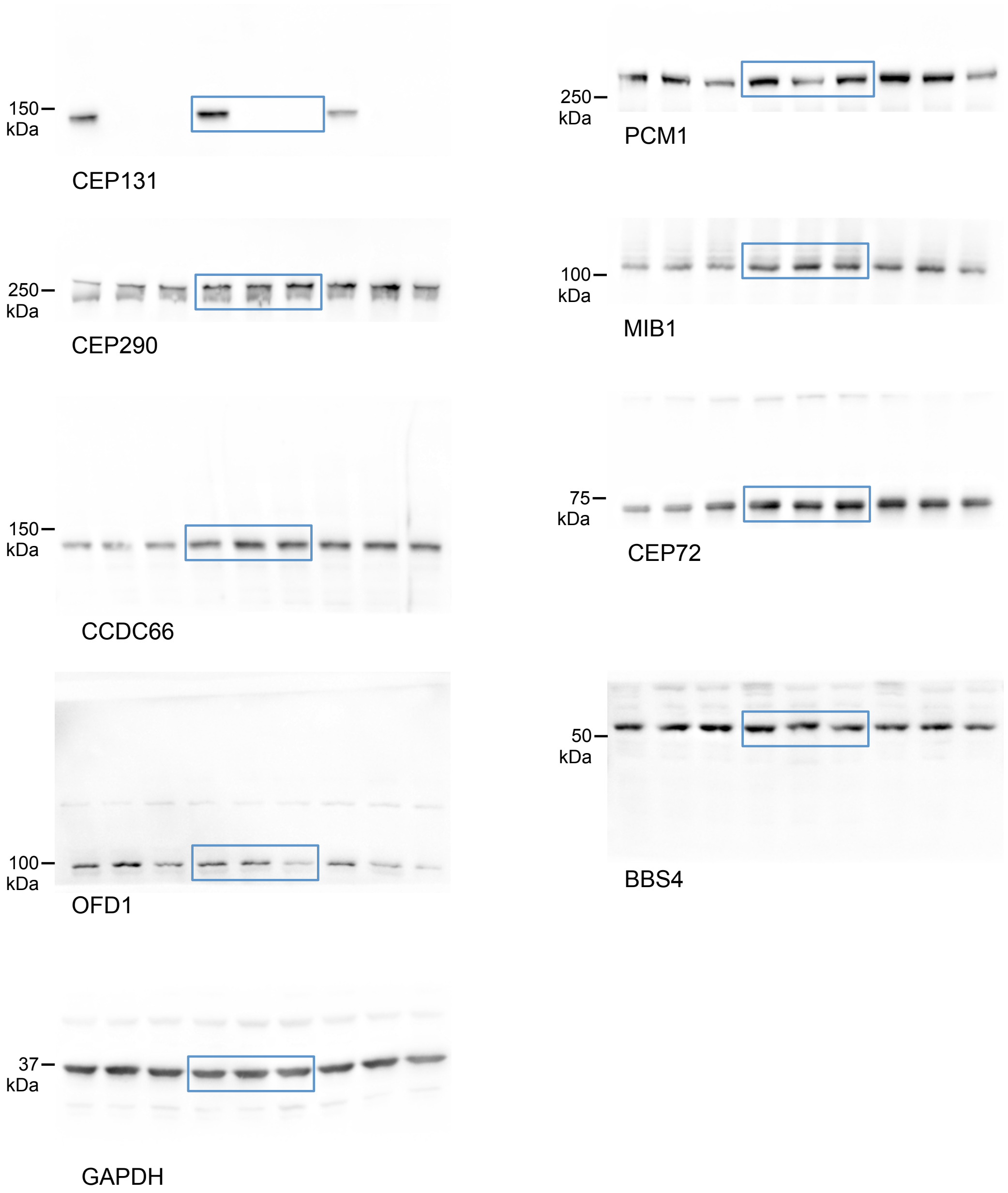

Figure 1h

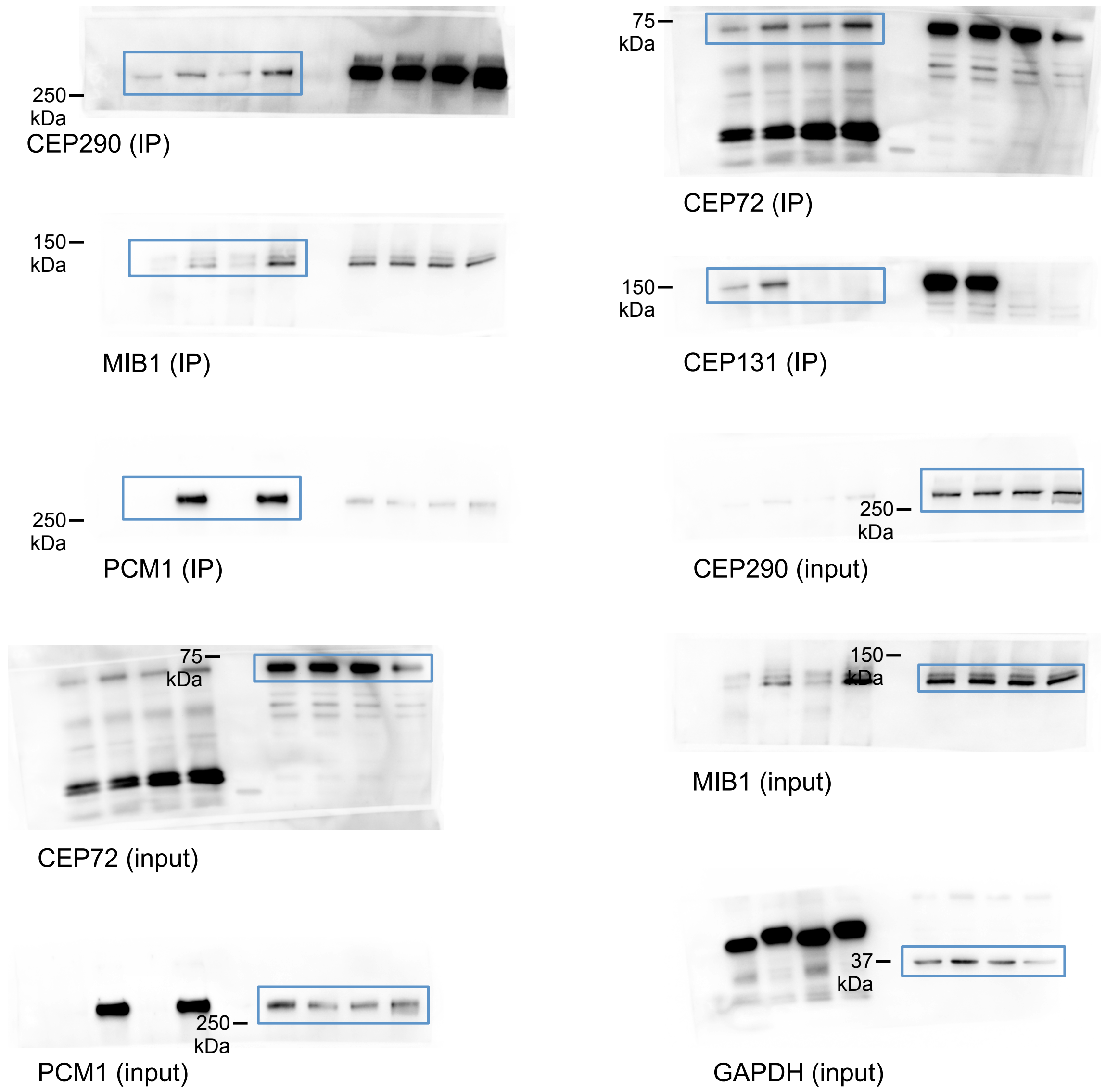

Figure 3g

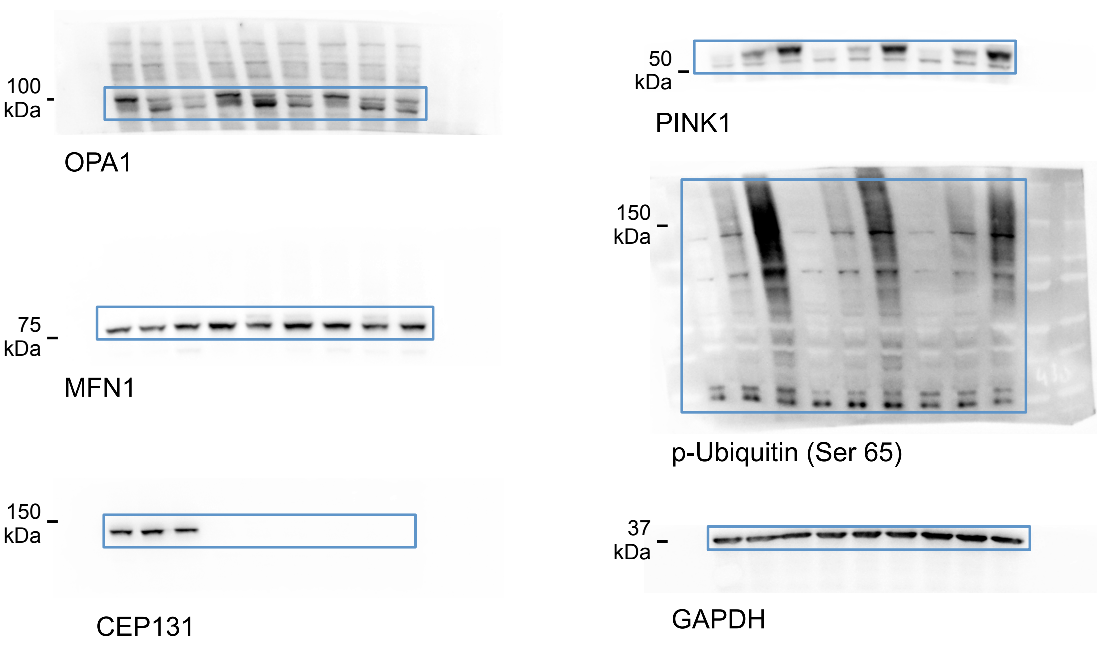

Figure 4c

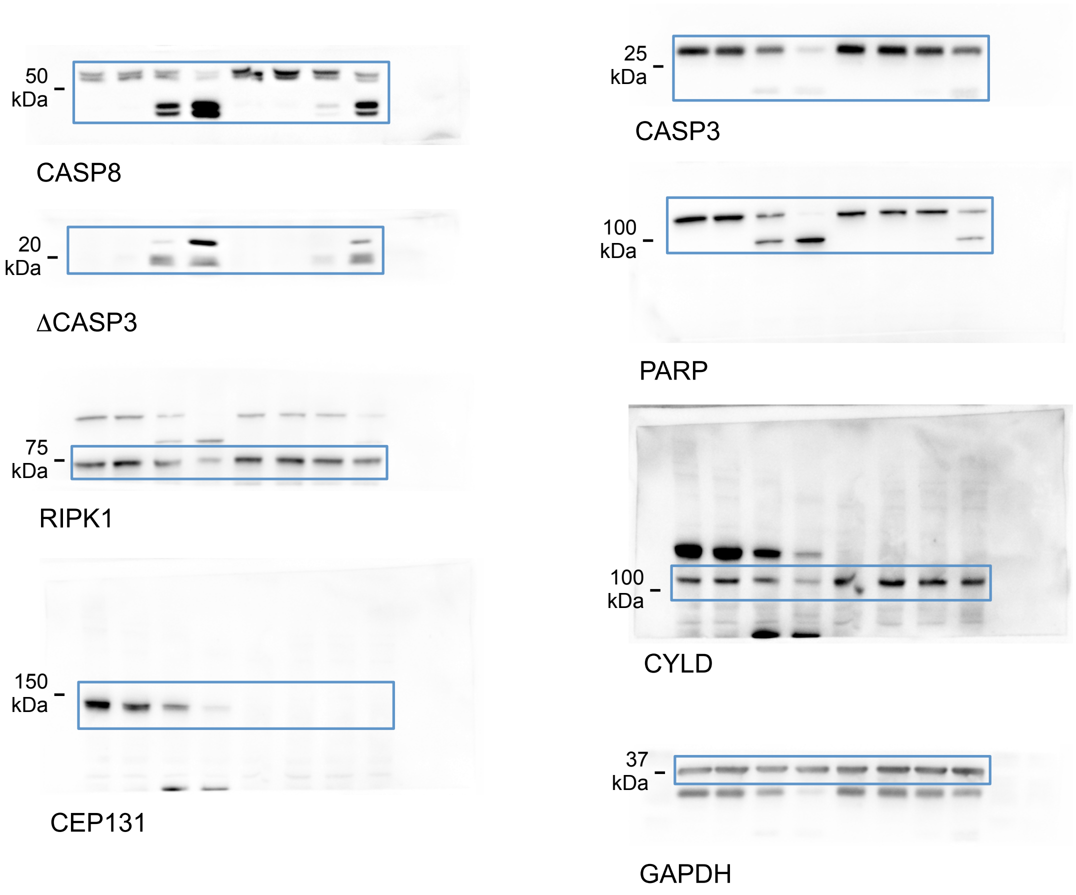

Figure 4g

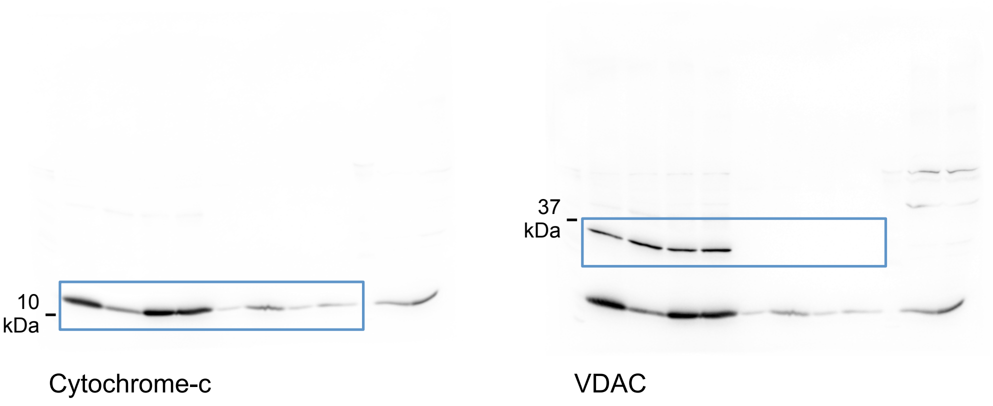

Supplementary Figure 1e

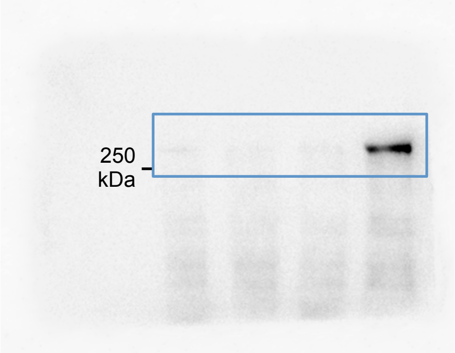

pATM

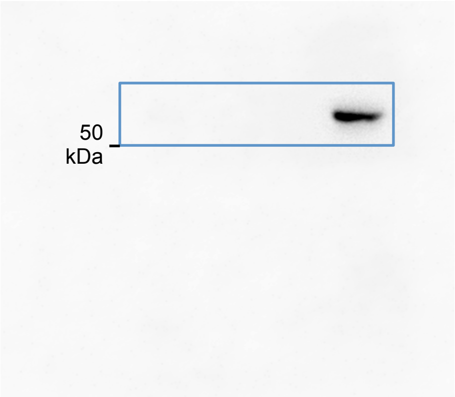

pCHK2

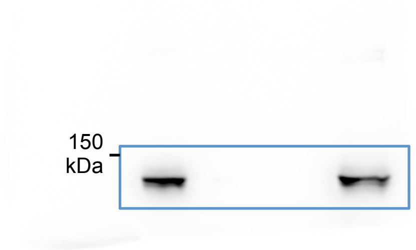

CEP131

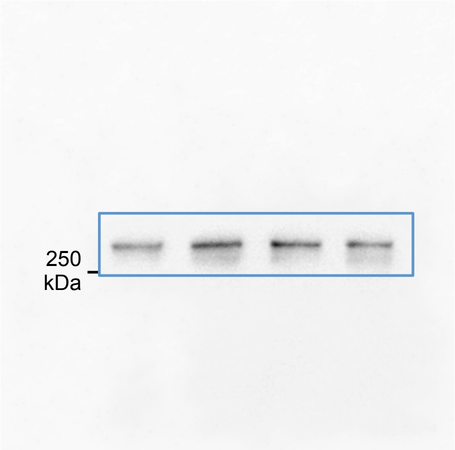

ATM

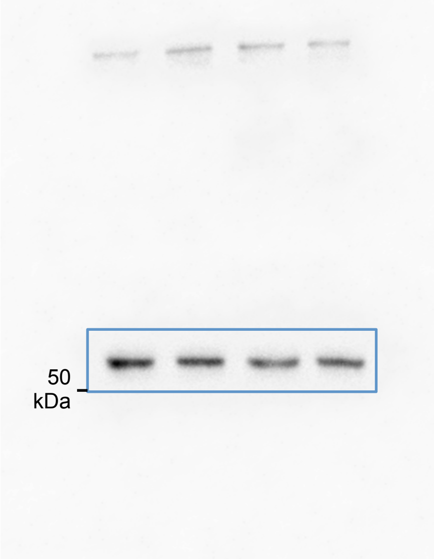

CHK2

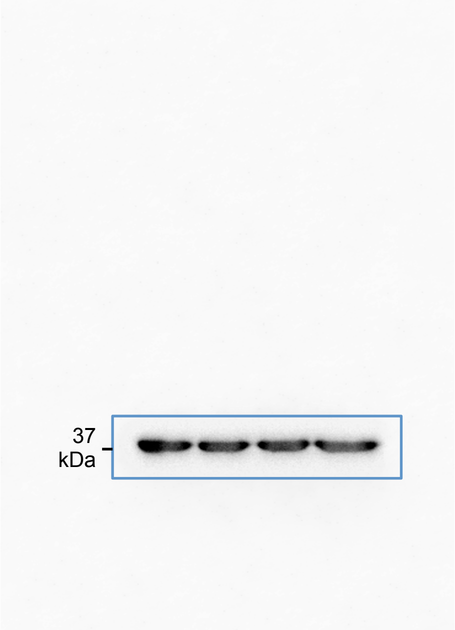

GAPDH

Supplementary Figure 1f

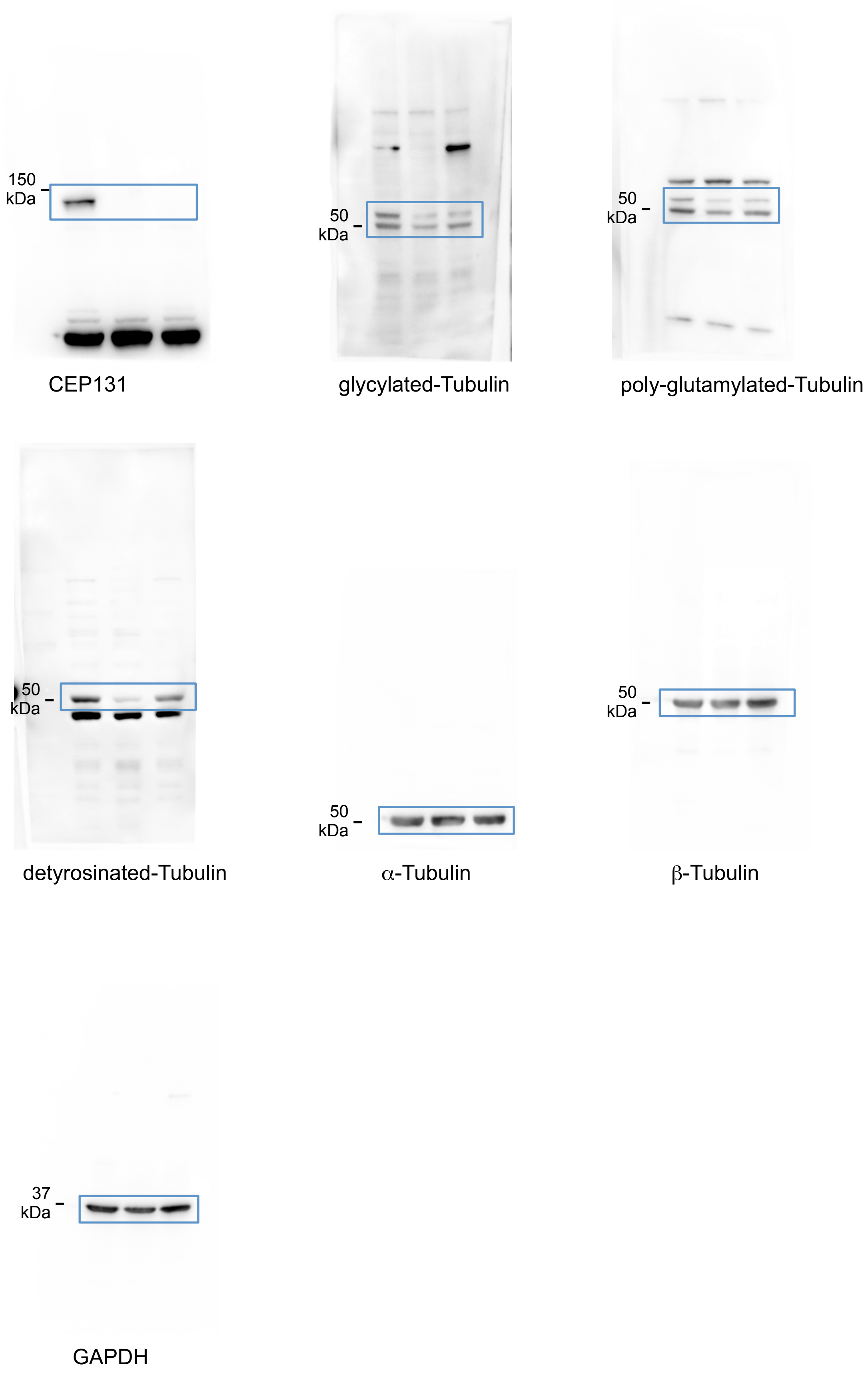

Supplementary Figure 2c

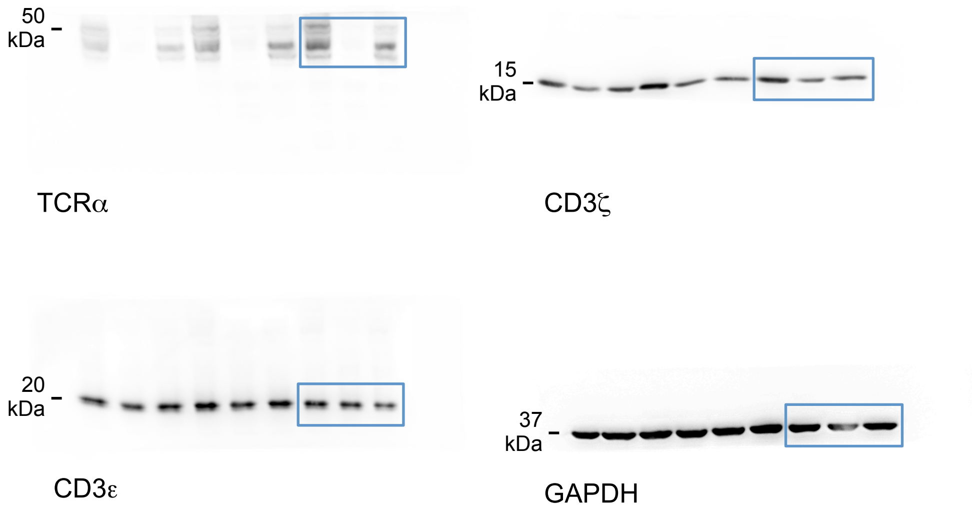

Supplementary Figure 2i

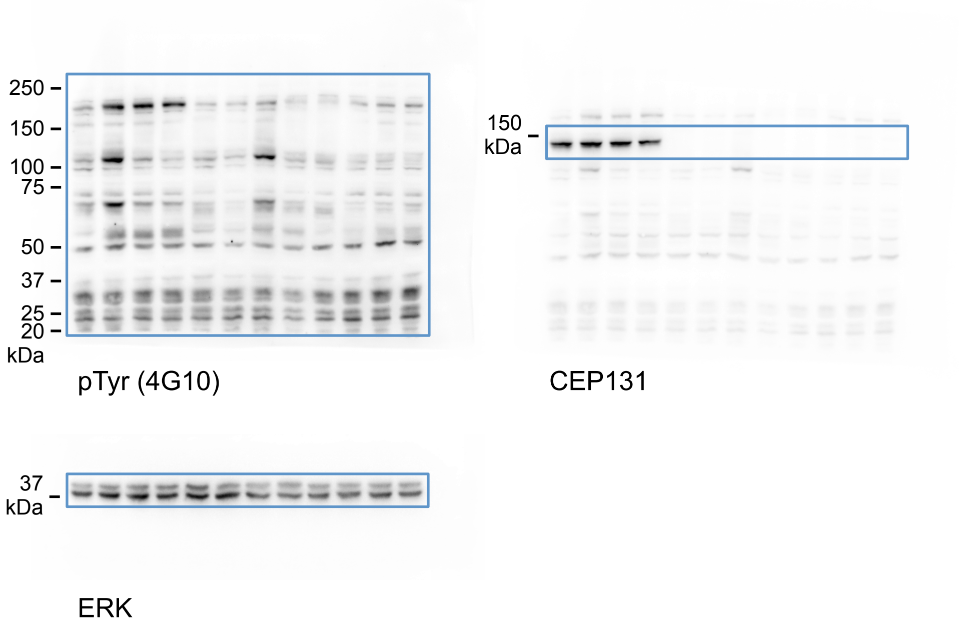

Supplementary Figure 2 j (anti-CD3/CD28)

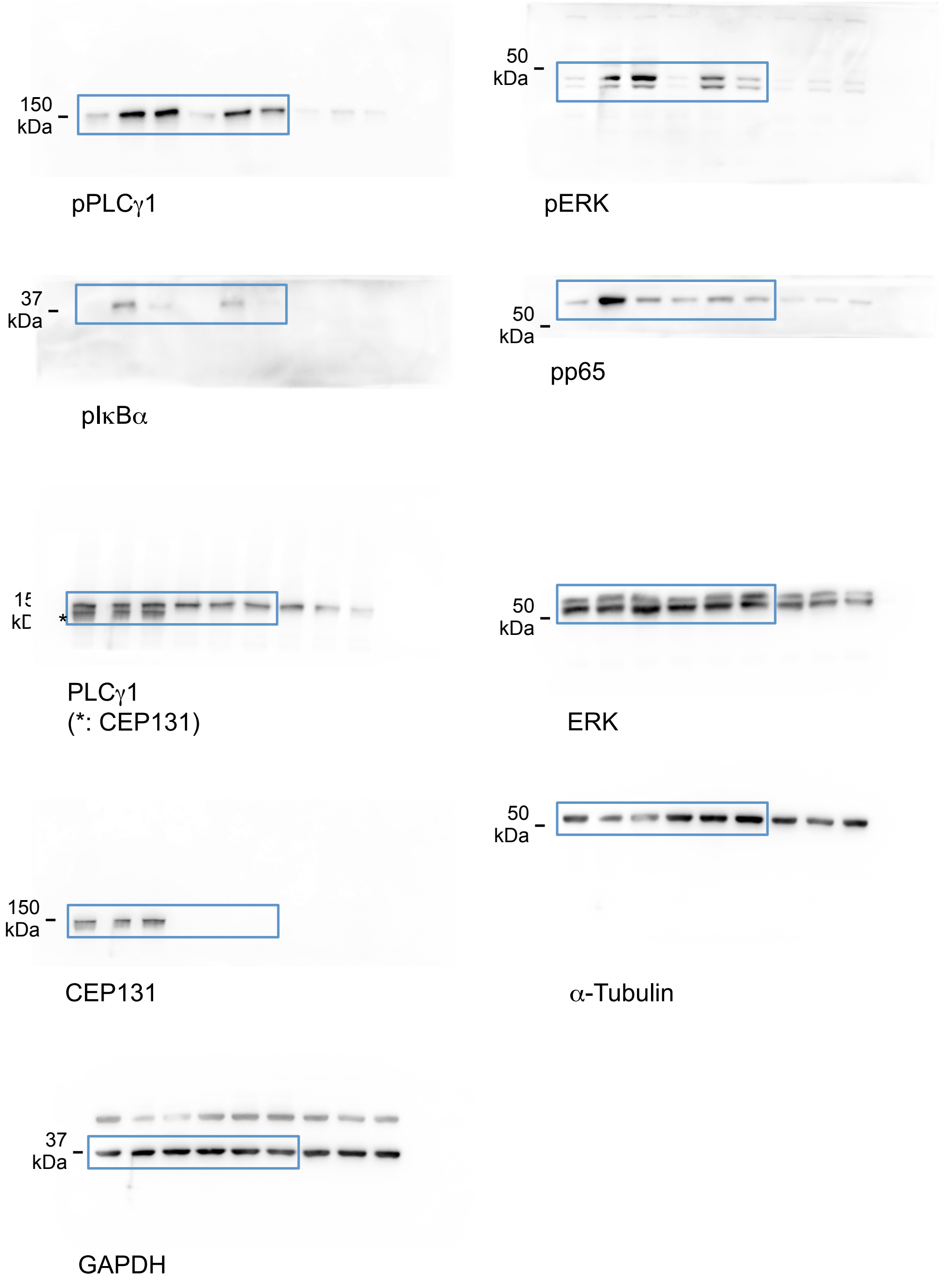

Supplementary Figure 2 j (PMA/ionomycin)

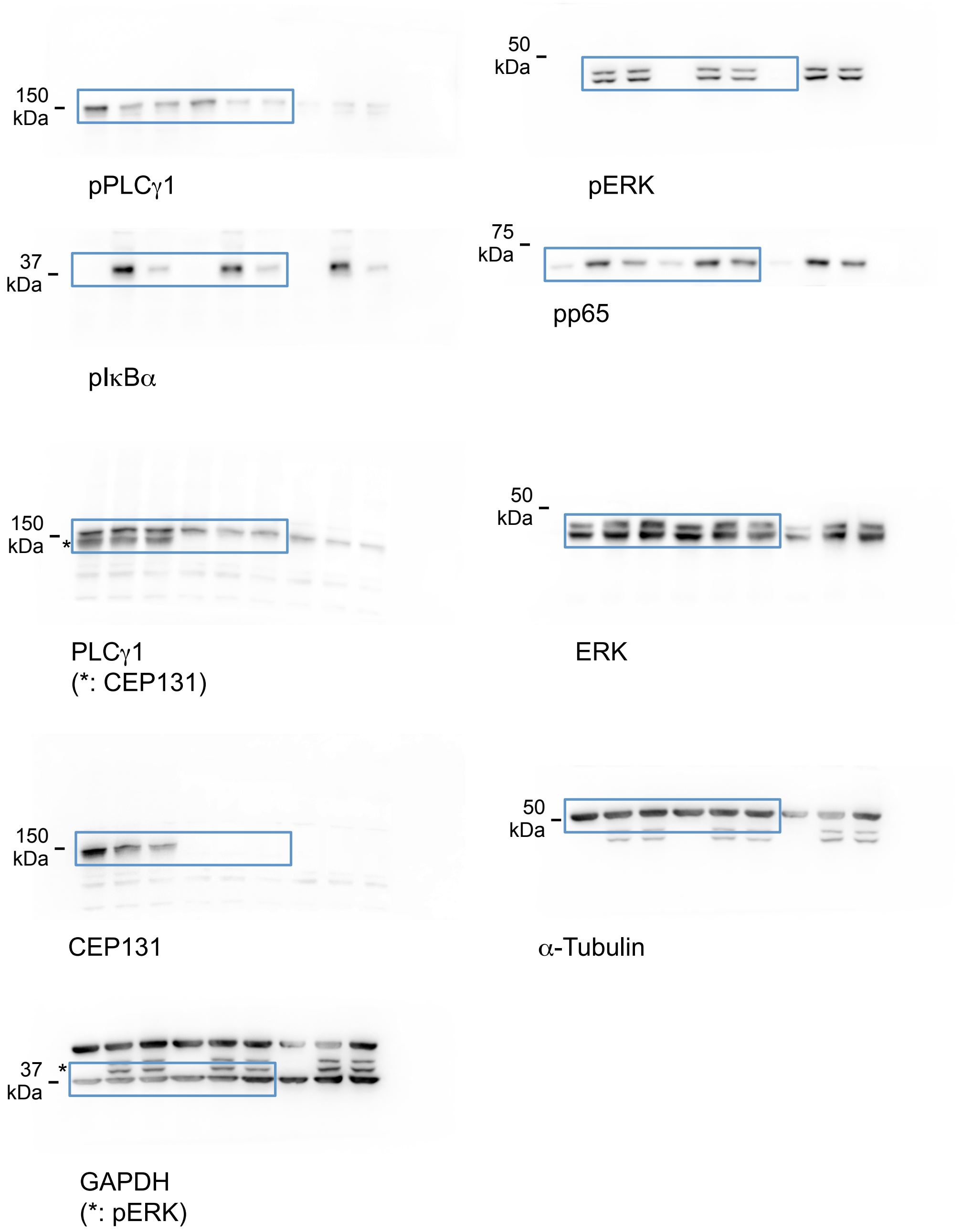

Supplementary Figure 3e

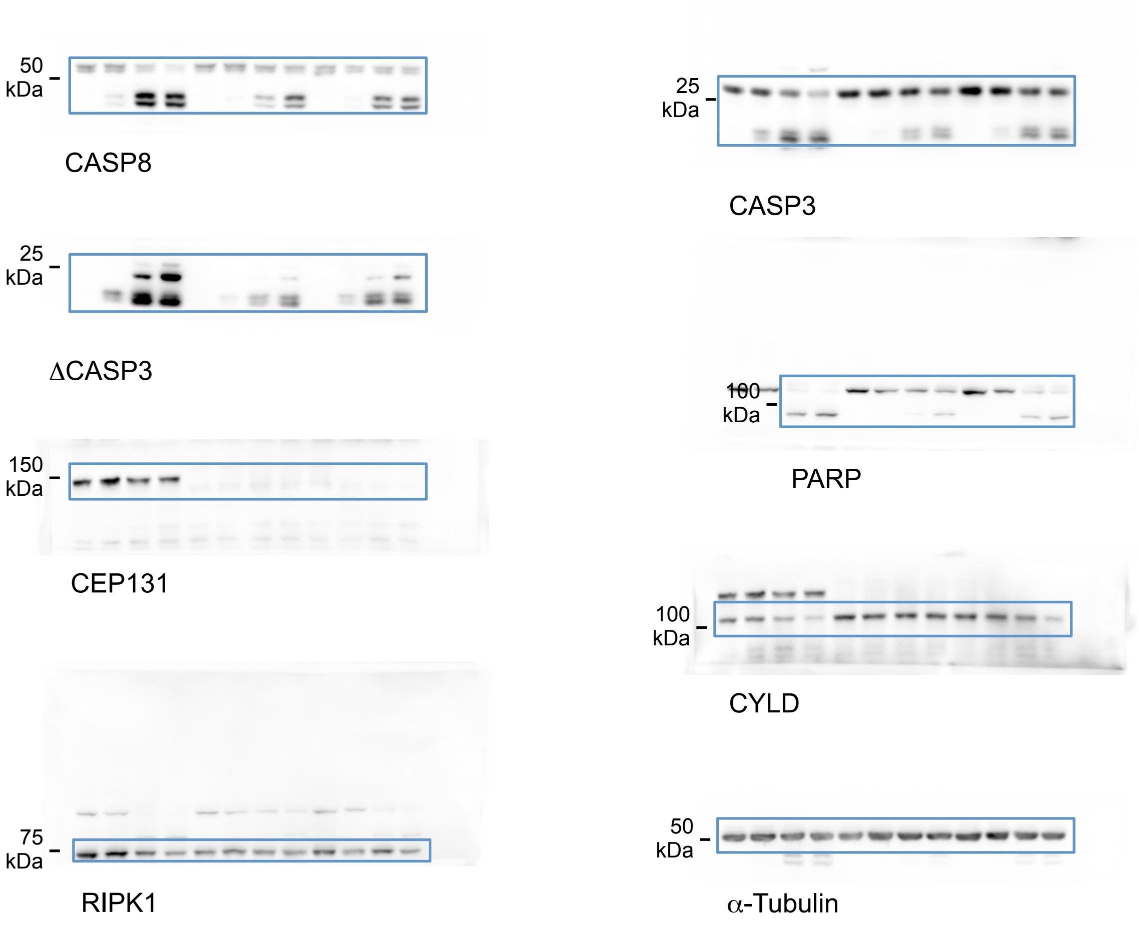

Supplementary Figure 3g

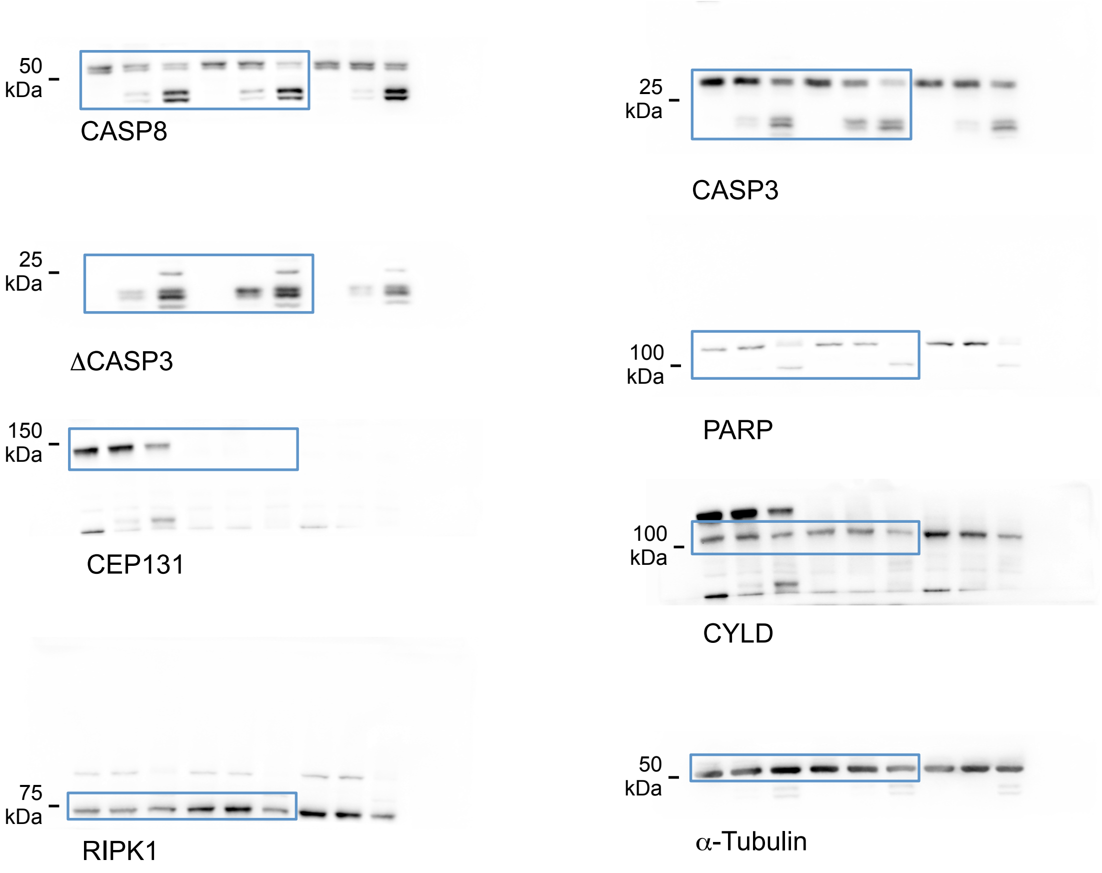

Supplementary Figure 4b

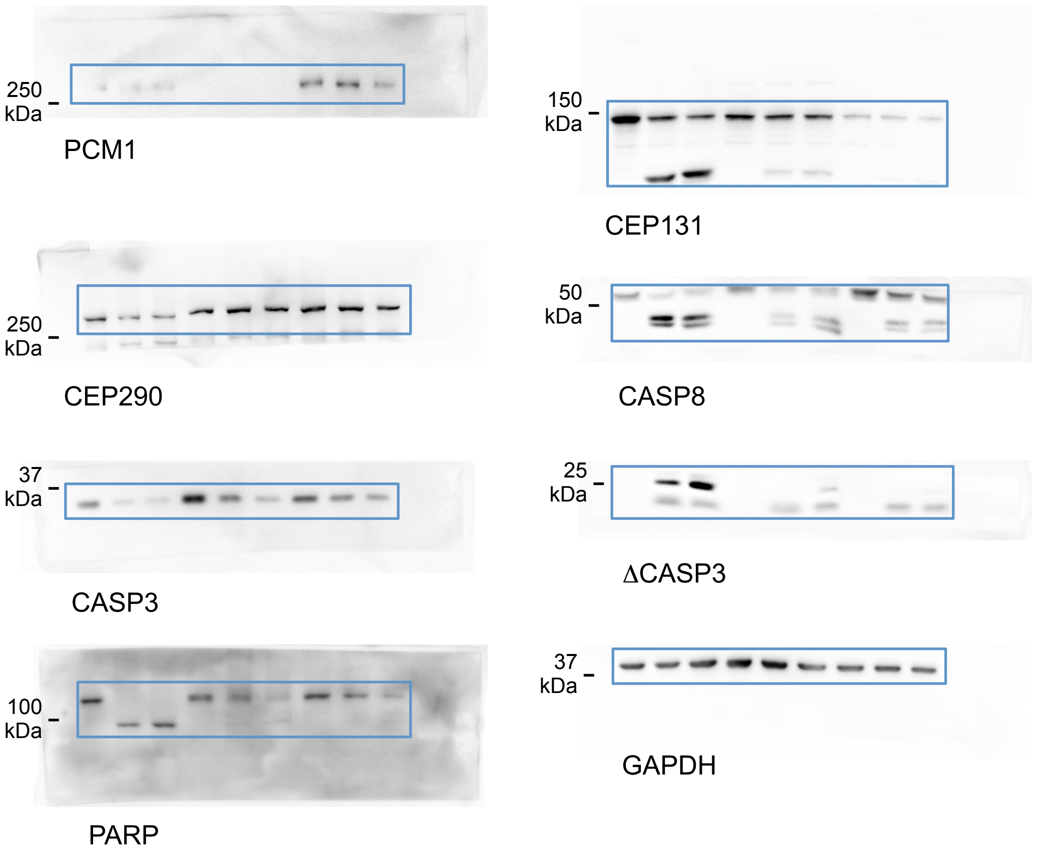

Supplementary Figure 4d

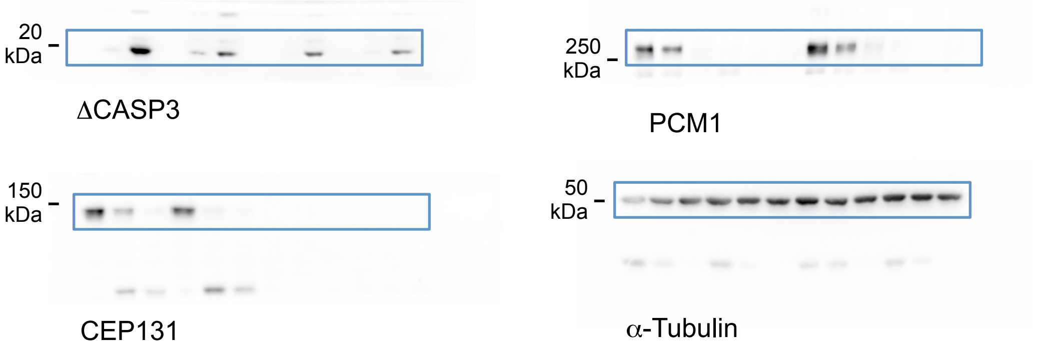

Supplementary Figure 5a

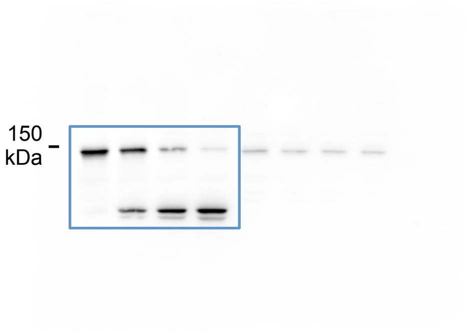

CEP131

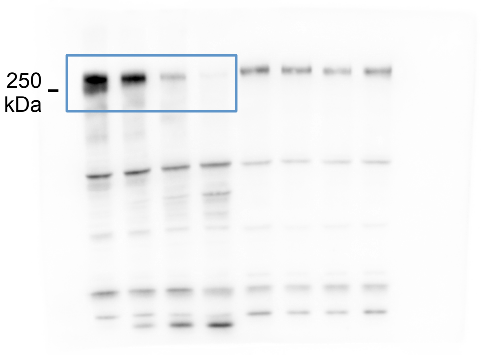

PCM1

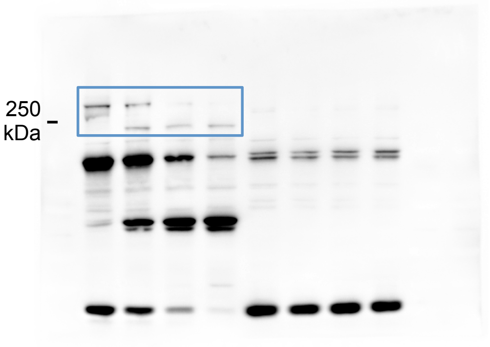

CEP290

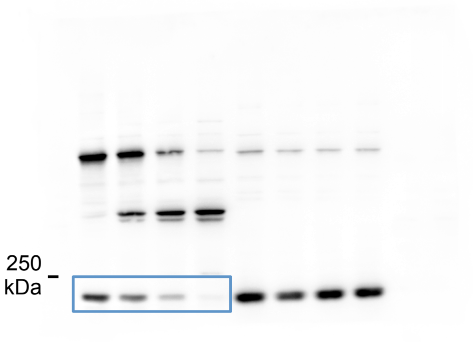

CASP3

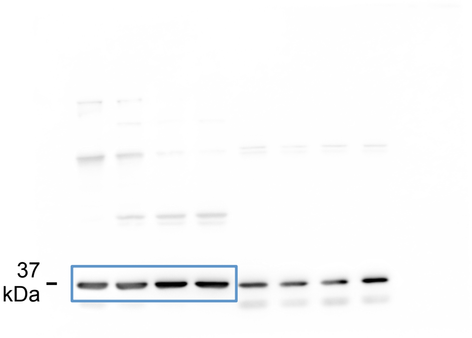

GAPDH

Supplementary Figure 5b

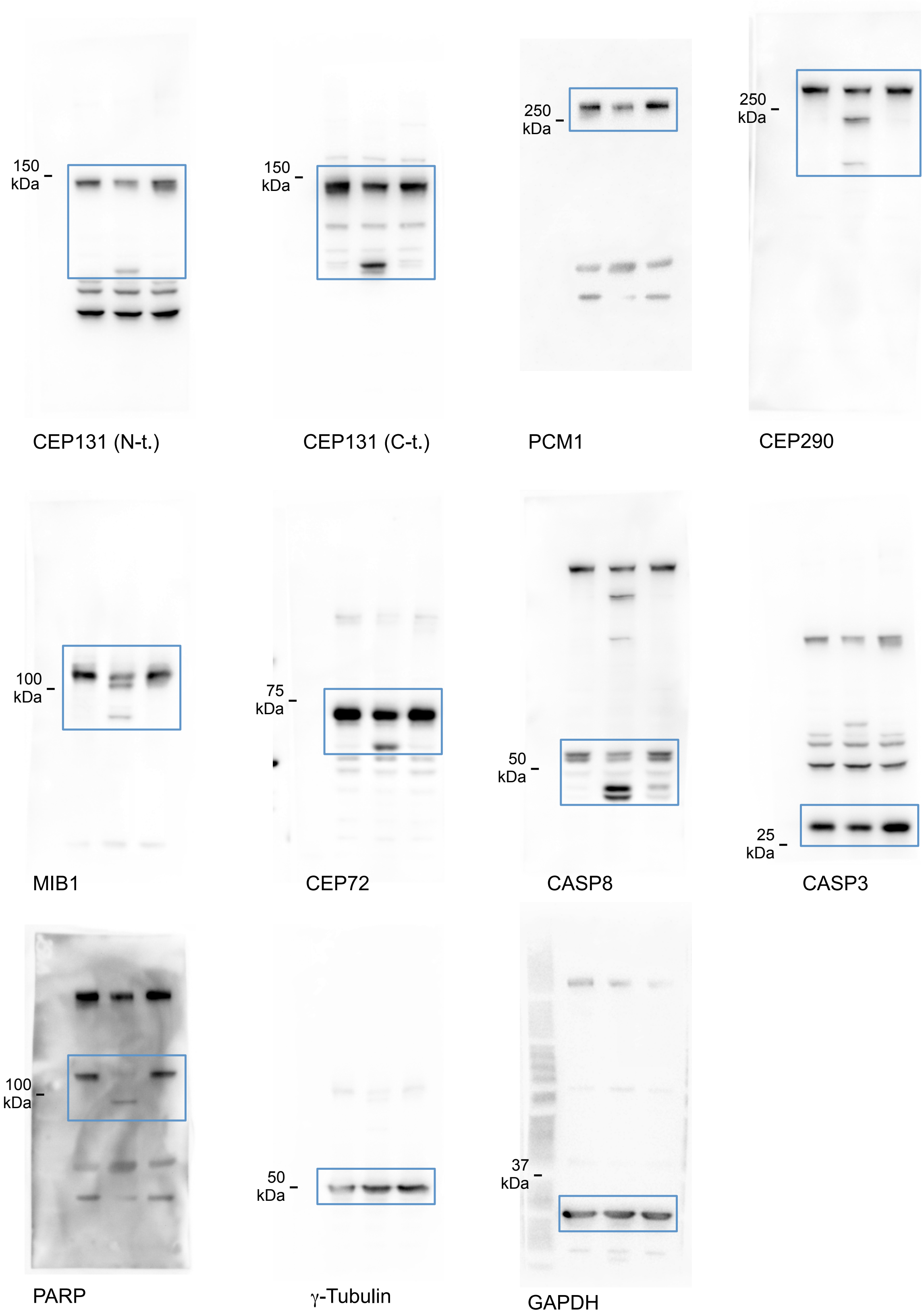

Supplementary Figure 5c

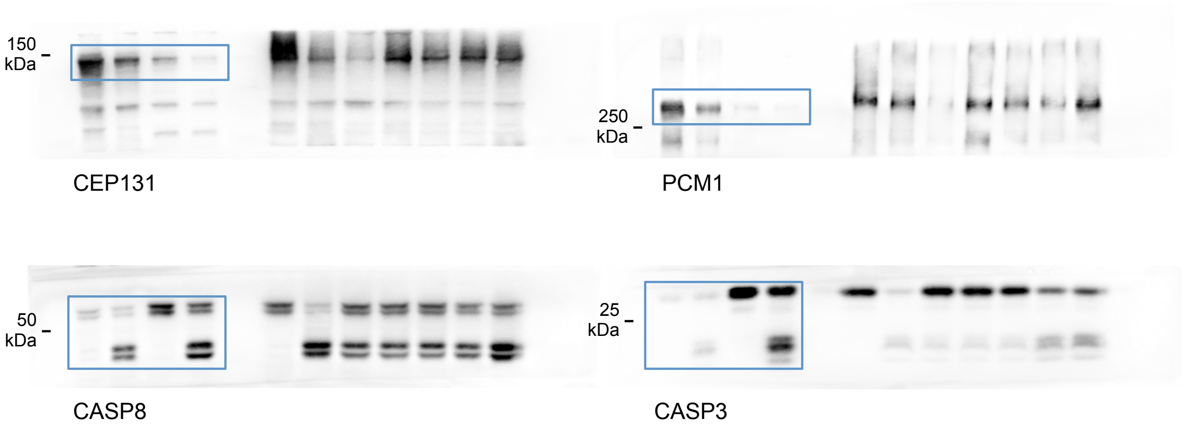

Supplementary Figure 5e

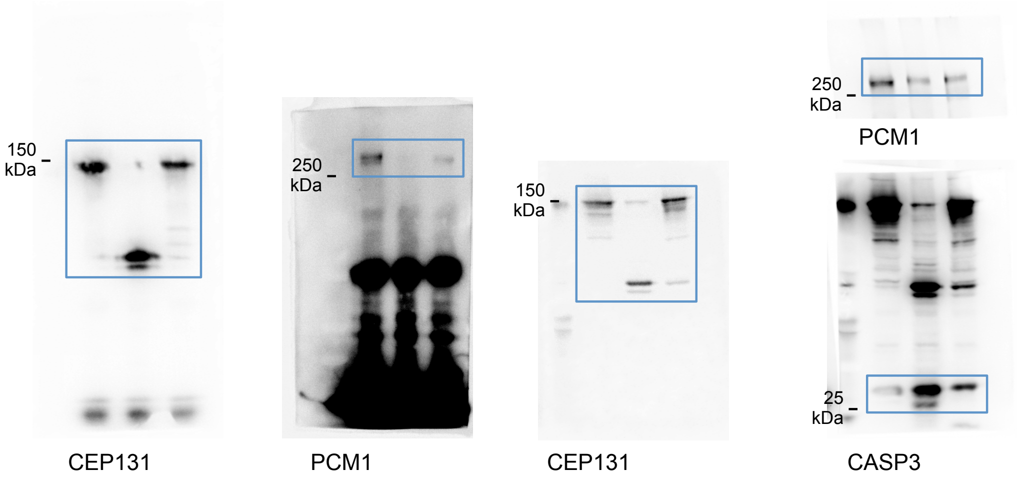

Supplementary Figure 5f

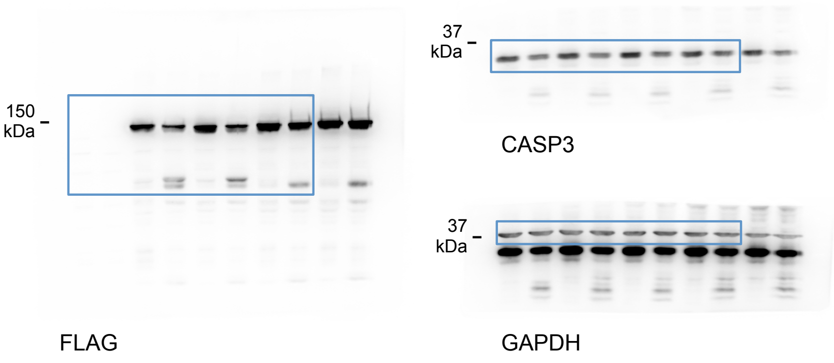

Supplementary Figure 5g

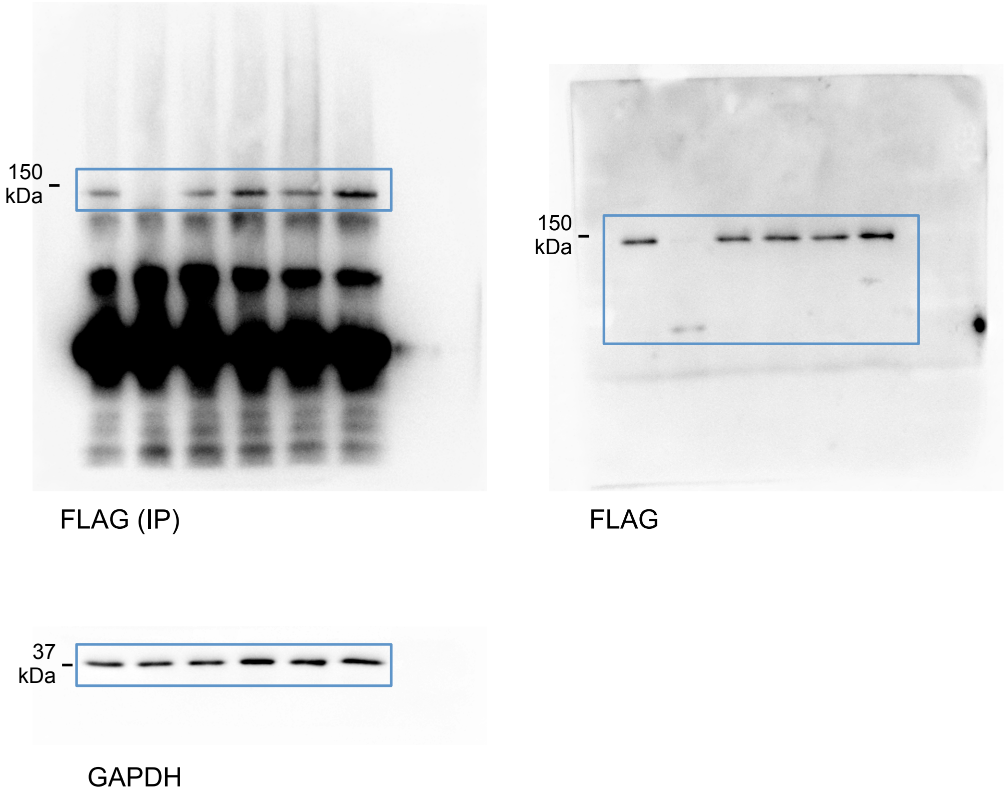

Appendix Table 1. Gene list of in silico predicted off-targets. Related to Fig. 1.

sgRNA	In silico predicted number of off-targets (0-4 mismatches)	Off-targets of 1, 2, and 3 mismatches
CACCGAAGA TCCAACAGC ACCACGC	0 mismatch: 0<br>1 mismatch: 0<br>2 mismatches: 2<br>3 mismatches: 5<br>4 mismatches: 90	2: intergenic: RP11-347I19.8-AC084018.1<br>2: intergenic: snoU13-RP11-305P14.1<br>3: intergenic: NINJ2-RP5-1154L15.2<br>3: intergenic: SPCS3-RP11-313E19.1<br>3: intron: LINGO1<br>3: intron: NTRK2<br>3: exon: LONRF3

Appendix Table 2. Gene list of qPCR Array. Related to Figure S2.

**Unigene**	**Refseq**	**Symbol**	**Description**
Hs.441047	NM_001124	ADM	Adrenomedullin
Hs.19383	NM_000029	AGT	Angiotensinogen (serpin peptidase inhibitor, clade A, member 8)
Hs.525622	NM_005163	AKT1	V-akt murine thymoma viral oncogene homolog 1
Hs.499886	NM_000382	ALDH3A2	Aldehyde dehydrogenase 3 family, member A2
Hs.227817	NM_004049	BCL2A1	BCL2-related protein A1
Hs.516966	NM_138578	BCL2L1	BCL2-like 1
Hs.696238	NM_001166	BIRC2	Baculoviral IAP repeat containing 2
Hs.127799	NM_001165	BIRC3	Baculoviral IAP repeat containing 3
Hs.529053	NM_000064	C3	Complement component 3
Hs.54460	NM_002986	CCL11	Chemokine (C-C motif) ligand 11
Hs.303649	NM_002982	CCL2	Chemokine (C-C motif) ligand 2
Hs.534347	NM_002990	CCL22	Chemokine (C-C motif) ligand 22
Hs.514821	NM_002985	CCL5	Chemokine (C-C motif) ligand 5
Hs.523852	NM_053056	CCND1	Cyclin D1
Hs.450802	NM_000579	CCR5	Chemokine (C-C motif) receptor 5
Hs.472860	NM_001250	CD40	CD40 molecule, TNF receptor superfamily member 5
Hs.208854	NM_001781	CD69	CD69 molecule
Hs.838	NM_005191	CD80	CD80 molecule
Hs.595133	NM_004233	CD83	CD83 molecule
Hs.370771	NM_000389	CDKN1A	Cyclin-dependent kinase inhibitor 1A (p21, Cip1)
Hs.69771	NM_001710	CFB	Complement factor B
Hs.173894	NM_000757	CSF1	Colony stimulating factor 1 (macrophage)
Hs.1349	NM_000758	CSF2	Colony stimulating factor 2 (granulocyte-macrophage)
Hs.592192	NM_000395	CSF2RB	Colony stimulating factor 2 receptor, beta, low-affinity (granulocyte-macrophage)
Hs.2233	NM_000759	CSF3	Colony stimulating factor 3 (granulocyte)
Hs.789	NM_001511	CXCL1	Chemokine (C-X-C motif) ligand 1 (melanoma growth stimulating activity, alpha)
Hs.632586	NM_001565	CXCL10	Chemokine (C-X-C motif) ligand 10
Hs.75765	NM_002089	CXCL2	Chemokine (C-X-C motif) ligand 2
Hs.77367	NM_002416	CXCL9	Chemokine (C-X-C motif) ligand 9
Hs.488293	NM_005228	EGFR	Epidermal growth factor receptor
Hs.1395	NM_000399	EGR2	Early growth response 2
Hs.62192	NM_001993	F3	Coagulation factor III (thromboplastin, tissue factor)
Hs.654450	NM_000132	F8	Coagulation factor VIII, procoagulant component
Hs.667309	NM_000043	FAS	Fas (TNF receptor superfamily, member 6)
Hs.2007	NM_000639	FASLG	Fas ligand (TNF superfamily, member 6)
Hs.110571	NM_015675	GADD45B	Growth arrest and DNA-damage-inducible, beta
Hs.643447	NM_000201	ICAM1	Intercellular adhesion molecule 1
Hs.93177	NM_002176	IFNB1	Interferon, beta 1, fibroblast
Hs.856	NM_000619	IFNG	Interferon, gamma
Hs.674	NM_002187	IL12B	Interleukin 12B (natural killer cell stimulatory factor 2, cytotoxic lymphocyte maturation factor 2, p40)
Hs.168132	NM_000585	IL15	Interleukin 15

Hs.1722	NM_000575	IL1A	Interleukin 1, alpha
Hs.126256	NM_000576	IL1B	Interleukin 1, beta
Hs.25333	NM_004633	IL1R2	Interleukin 1 receptor, type II
Hs.81134	NM_000577	IL1RN	Interleukin 1 receptor antagonist
Hs.89679	NM_000586	IL2	Interleukin 2
Hs.231367	NM_000417	IL2RA	Interleukin 2 receptor, alpha
Hs.73917	NM_000589	IL4	Interleukin 4
Hs.654458	NM_000600	IL6	Interleukin 6 (interferon, beta 2)
Hs.624	NM_000584	CXCL8	Interleukin 8
Hs.700350	NM_000207	INS	Insulin
Hs.436061	NM_002198	IRF1	Interferon regulatory factor 1
Hs.36	NM_000595	LTA	Lymphotoxin alpha (TNF superfamily, member 1)
Hs.376208	NM_002341	LTB	Lymphotoxin beta (TNF superfamily, member 3)
Hs.463978	NM_002758	MAP2K6	Mitogen-activated protein kinase kinase 6
Hs.297413	NM_004994	MMP9	Matrix metallopeptidase 9 (gelatinase B, 92kDa gelatinase, 92kDa type IV collagenase)
Hs.202453	NM_002467	MYC	V-myc myelocytomatosis viral oncogene homolog (avian)
Hs.82116	NM_002468	MYD88	Myeloid differentiation primary response gene (88)
Hs.592142	NM_181659	NCOA3	Nuclear receptor coactivator 3
Hs.618430	NM_003998	NFKB1	Nuclear factor of kappa light polypeptide gene enhancer in B-cells 1
Hs.73090	NM_002502	NFKB2	Nuclear factor of kappa light polypeptide gene enhancer in B-cells 2 (p49/p100)
Hs.81328	NM_020529	NFKBIA	Nuclear factor of kappa light polypeptide gene enhancer in B-cells inhibitor, alpha
Hs.406515	NM_000903	NQO1	NAD(P)H dehydrogenase, quinone 1
Hs.563344	NM_006186	NR4A2	Nuclear receptor subfamily 4, group A, member 2
Hs.1976	NM_002608	PDGFB	Platelet-derived growth factor beta polypeptide
Hs.77274	NM_002658	PLAU	Plasminogen activator, urokinase
Hs.196384	NM_000963	PTGS2	Prostaglandin-endoperoxide synthase 2 (prostaglandin G/H synthase and cyclooxygenase)
Hs.633256	NM_002908	REL	V-rel reticuloendotheliosis viral oncogene homolog (avian)
Hs.502875	NM_021975	RELA	V-rel reticuloendotheliosis viral oncogene homolog A (avian)
Hs.654402	NM_006509	RELB	V-rel reticuloendotheliosis viral oncogene homolog B
Hs.82848	NM_000450	SELE	Selectin E
Hs.73800	NM_003005	SELP	Selectin P (granule membrane protein 140kDa, antigen CD62)
Hs.167317	NM_003081	SNAP25	Synaptosomal-associated protein, 25kDa
Hs.487046	NM_000636	SOD2	Superoxide dismutase 2, mitochondrial
Hs.642990	NM_007315	STAT1	Signal transducer and activator of transcription 1, 91kDa
Hs.463059	NM_003150	STAT3	Signal transducer and activator of transcription 3 (acute-phase response factor)
Hs.595276	NM_012448	STAT5B	Signal transducer and activator of transcription 5B
Hs.241570	NM_000594	TNF	Tumor necrosis factor
Hs.256278	NM_001066	TNFRSF1B	Tumor necrosis factor receptor superfamily, member 1B
Hs.478275	NM_003810	TNFSF10	Tumor necrosis factor (ligand) superfamily, member 10
Hs.437460	NM_000546	TP53	Tumor protein p53
Hs.522506	NM_021138	TRAF2	TNF receptor-associated factor 2
Hs.109225	NM_001078	VCAM1	Vascular cell adhesion molecule 1

Hs.356076	NM_001167	XIAP	X-linked inhibitor of apoptosis
Hs.520640	NM_001101	ACTB	Actin, beta
Hs.534255	NM_004048	B2M	Beta-2-microglobulin
Hs.592355	NM_002046	GAPDH	Glyceraldehyde-3-phosphate dehydrogenase
Hs.412707	NM_000194	HPRT1	Hypoxanthine phosphoribosyltransferase 1
Hs.546285	NM_001002	RPLP0	Ribosomal protein, large, P0
