# Supplementary material for: The centrosomal protein 131 participates in the regulation of mitochondrial apoptosis
Source: Commun Biol. 2023 Dec 15;6:1271. doi: 10.1038/s42003-023-05676-3 (PMC10724242; doi:10.1038/s42003-023-05676-3)
Supplement: Supplementary file 3 — Description of additional supplementary files [file 42003_2023_5676_MOESM3_ESM.docx]

**Description of Additional Supplementary Files**

File name: Supplementary Data 1

Description: Numerical source data for Figs. 1-4, Supplementary Figs. 1-7, and Appendix Tables 1 and 2.
