# Supplementary material for: The centrosomal protein 131 participates in the regulation of mitochondrial apoptosis
Source: Commun Biol. 2023 Dec 15;6:1271. doi: 10.1038/s42003-023-05676-3 (PMC10724242; doi:10.1038/s42003-023-05676-3)
Supplement: Supplementary file 5 — Reporting summary [file 42003_2023_5676_MOESM5_ESM.pdf]

nature portfolio

Corresponding author(s): BIDERE Nicolas

Last updated by author(s): Sep 26, 2023

Reporting Summary

Nature Portfolio wishes to improve the reproducibility of the work that we publish. This form provides structure for consistency and transparency in reporting. For further information on Nature Portfolio policies, see our Editorial Policies and the Editorial Policy Checklist.

Statistics

For all statistical analyses, confirm that the following items are present in the figure legend, table legend, main text, or Methods section.

n/a	Confirmed	
☐	☒	The exact sample size (*n*) for each experimental group/condition, given as a discrete number and unit of measurement
☐	☒	A statement on whether measurements were taken from distinct samples or whether the same sample was measured repeatedly
☐	☒	The statistical test(s) used AND whether they are one- or two-sided<br>*Only common tests should be described solely by name; describe more complex techniques in the Methods section.*
☐	☒	A description of all covariates tested
☐	☒	A description of any assumptions or corrections, such as tests of normality and adjustment for multiple comparisons
☐	☒	A full description of the statistical parameters including central tendency (e.g. means) or other basic estimates (e.g. regression coefficient) AND variation (e.g. standard deviation) or associated estimates of uncertainty (e.g. confidence intervals)
☒	☐	For null hypothesis testing, the test statistic (e.g. *F*, *t*, *r*) with confidence intervals, effect sizes, degrees of freedom and *P* value noted<br>*Give P values as exact values whenever suitable.*
☒	☐	For Bayesian analysis, information on the choice of priors and Markov chain Monte Carlo settings
☒	☐	For hierarchical and complex designs, identification of the appropriate level for tests and full reporting of outcomes
☒	☐	Estimates of effect sizes (e.g. Cohen's *d*, Pearson's *r*), indicating how they were calculated

Our web collection on statistics for biologists contains articles on many of the points above.

Software and code

Policy information about availability of computer code

Data collection	GraphPad Prism 8
Data analysis	GraphPad Prism 8

For manuscripts utilizing custom algorithms or software that are central to the research but not yet described in published literature, software must be made available to editors and reviewers. We strongly encourage code deposition in a community repository (e.g. GitHub). See the Nature Portfolio guidelines for submitting code & software for further information.

Data

Policy information about availability of data

All manuscripts must include a data availability statement. This statement should provide the following information, where applicable:

- Accession codes, unique identifiers, or web links for publicly available datasets
- A description of any restrictions on data availability
- For clinical datasets or third party data, please ensure that the statement adheres to our policy

All data supporting the findings of this study are available within the paper and its Supplementary Information.

Research involving human participants, their data, or biological material

Policy information about studies with human participants or human data. See also policy information about sex, gender (identity/presentation), and sexual orientation and race, ethnicity and racism.

Reporting on sex and gender	NA
Reporting on race, ethnicity, or other socially relevant groupings	NA
Population characteristics	NA
Recruitment	NA
Ethics oversight	NA

Note that full information on the approval of the study protocol must also be provided in the manuscript.

Field-specific reporting

Please select the one below that is the best fit for your research. If you are not sure, read the appropriate sections before making your selection.

☒ Life sciences ☐ Behavioural & social sciences ☐ Ecological, evolutionary & environmental sciences

For a reference copy of the document with all sections, see nature.com/documents/nr-reporting-summary-flat.pdf

Life sciences study design

All studies must disclose on these points even when the disclosure is negative.

Sample size	NA
Data exclusions	NA
Replication	NA
Randomization	NA
Blinding	NA

Reporting for specific materials, systems and methods

We require information from authors about some types of materials, experimental systems and methods used in many studies. Here, indicate whether each material, system or method listed is relevant to your study. If you are not sure if a list item applies to your research, read the appropriate section before selecting a response.

Materials & experimental systems

n/a	Involved in the study	
☐	☒	Antibodies
☐	☒	Eukaryotic cell lines
☒	☐	Palaeontology and archaeology
☒	☐	Animals and other organisms
☒	☐	Clinical data
☒	☐	Dual use research of concern
☒	☐	Plants

Methods

n/a	Involved in the study	
☒	☐	ChIP-seq
☐	☒	Flow cytometry
☒	☐	MRI-based neuroimaging

Antibodies

Antibodies used	Western Blotting. Antibodies specific for the following proteins were purchased from Santa Cruz Biotechnology: CD3ε (SC-1179), CD3ζ (SC-1239), CEP290 (SC-390462), CYLD (SC-137139), DRP1 (SC-271583), GAPDH (SC-47724), MIB1 (SC-393811), PARK8 (sc32282), PARP (SC-8007), PCM1 (SC-398365), TBK1 (SC-73115), TCRα (SC-51571), γ-Tubulin (SC-51715). Antibodies against ATM (2873), caspase-3 (9662), cleaved caspase-3 (9664), caspase-8 (9746L), CHK2 (3440), DYKDDDDK Tag (FLAG-M2; 8146), LC3B (3868), MFN1

(14739), pATM (5883), pCHK2 (2661), pERK (9106S), pIκBα (S32/36) (9246), PLCγ1 (5690), pPLCγ1 (14008), pp65 (S536) (11/17), and RIPK1 (3493) were purchased from Cell Signaling Technology. Antibodies specific for the following proteins were obtained from Proteintech: BBS4 (12766-1-AP), CEP131 (C-ter, 25735-1-AP), cytochrome c (10993-1-AP), GABARAPL1 (11010-1-AP) α-Tubulin (66031-1-1g), β-Tubulin (66240-1-1g). Antibodies against CCDC66 (A303-339A; Bethyl), CEP72 (A301-297A, Bethyl), CEP131 (N-ter, A301-415A, Bethyl), CEP290 (A301-659A, Bethyl), ERK (ab218017, Abcam), glycylated tubulin (AG-25B-0034-C100, Coger), polyglutamate chain (polyE, AG-25B-0030-C050, Coger), polyglutamylation modification tubulin (AG-20B-0020-C100, Coger), PCM1 (ab72443, Abcam), OFD1 (HPA031103, Atlas antibodies), and phospho-tyrosine (OR-321X, Millipore) and phospho-ubiquitin Ser65 (ABS1513-I) were also used.
Immunofluorescence. The following antibodies were used: CEP72 (A301-297A, Bethyl), CEP131 (A301-415A, Bethyl), CEP290 (IHC-00365-2, Bethyl), cytochrome-c (SC-13561, Santa Cruz), MIB1 (M5948, Sigma-Aldrich), PCM1 (5213S, Cell Signaling), α-Tubulin (66031-1-1g, Proteintech), γ-Tubulin (T6557, Sigma-Aldrich).
Flow Cytometry. Specific antibodies were used: FITC anti-human TCR⍰/⍰ (BD Pharmingen, 347773), AlexaFluor 647 anti-human CD3⍰ (CD247, BD Pharmingen, 566651), FITC anti-human CD3⍰ (BD Pharmingen, 555332), APC anti-human CD28 (BD Pharmingen, 559770).

Validation: Commercial antibodies, used according to the manufacturer's recommendations.

Eukaryotic cell lines

Policy information about cell lines and Sex and Gender in Research

Cell line source(s)	Jurkat E6.1 T lymphocytes, HeLa cells, L929 cells, and HT-29 cells were purchased from American Type Culture Collection (ATCC).
Authentication	Commercial source
Mycoplasma contamination	negative for mycoplasma
Commonly misidentified lines (See ICLAC register)	NA

Flow Cytometry

Plots

Confirm that:

☒ The axis labels state the marker and fluorochrome used (e.g. CD4-FITC).

☒ The axis scales are clearly visible. Include numbers along axes only for bottom left plot of group (a 'group' is an analysis of identical markers).

☒ All plots are contour plots with outliers or pseudocolor plots.

☒ A numerical value for number of cells or percentage (with statistics) is provided.

Methodology

Sample preparation	Mitochondrial mass was measured after incubating cells with 12.5 nM of MitoTracker Green (MTG) for 1h at 37°C. For mitochondrial membrane potential, Tetramethylrhodamine, methyl ester (TMRM) was used at 0.1 µM for 30 min at 37°C before flow cytometry analysis. The staining of surface proteins was performed on living cells with antibodies incubated for 1h at 4°C. Intracellular staining was performed using BD Cytofix/Cytoperm Kit (BD Biosciences) following the manufacturer's protocol. For the CD3⍰ recycling experiment, cells were treated with 1 µM Phorbol 12,13-dibutyrate (PDBu) for 1 h at 37 °C, washed at 4 °C and put at 37 °C for 0-60 min, as previously described. CD3 expression at the surface was measured by flow cytometry at 5, 10, 15, 30, and 60 min, and normalized by total surface CD3⍰.
Instrument	FACS Calibur (BD Biosciences)
Software	Cell Quest
Cell population abundance	no sorting
Gating strategy	cells, except debris, were analyzed

☐ Tick this box to confirm that a figure exemplifying the gating strategy is provided in the Supplementary Information.
